# From Bench to Operating Room: A Comparative Study of Tensile and Puncture Tests in Self-Assembled Constructs with Clinical Feedback

**DOI:** 10.3390/bioengineering13091051

**Published:** 2026-09-10

**Authors:** Elissa Elia, Marilou Hardy, Riham Mira Bensalem, Yudai Sahuc, Yannick Rioux, David Brownell, Stéphane Chabaud, Julie Fradette, Stéphane Bolduc

**Affiliations:** 1 Department of Surgery, Faculty of Medicine, Université Laval, Quebec City, QC G1V 0A6, Canada; elissa.elia@crchudequebec.ulaval.ca (E.E.); marilou.hardy@crchudequebec.ulaval.ca (M.H.); yudai.sahuc@crchudequebec.ulaval.ca (Y.S.); david.brownell@crchudequebec.ulaval.ca (D.B.); julie.fradette@fmed.ulaval.ca (J.F.); 2 Centre de Recherche en Organogenèse Expérimentale de l’Université Laval/LOEX, Centre de Recherche du CHU de Québec-Université Laval, Axe Médecine Régénératrice, Quebec City, QC G1J 1Z4, Canada; yannick.rioux.1@ulaval.ca (Y.R.); stephane.chabaud@crchudequebec.ulaval.ca (S.C.); 3 Faculty of Pharmacy, Université Grenoble Alpes, 38700 Grenoble, France; mira.bensalem22@gmail.com; 4 Department of Mechanical Engineering, Faculty of Sciences and Engineering, Université Laval, Quebec City, QC G1V 0A6, Canada

**Keywords:** uniaxial tensile test, puncture test, mechanical properties, tissue engineering, self-assembly, urethral reconstruction

## Abstract

Urethral reconstruction is required for a variety of congenital and acquired conditions. Current grafting techniques rely on autologous tissues such as buccal mucosa, which are often limited by donor-site morbidity. Biomaterials could be an alternative. However, they may present immunological risks and inconsistent surgical outcomes. The tissue-engineering self-assembly approach, which uses autologous patient cells to generate tissue constructs without exogenous biomaterials, offers a promising alternative by improving graft integration. Such natural tissue-engineered constructs exhibit promising histological similarity to native urethral tissue and hold potential for clinical application. However, graft selection for surgery remains subjective, lacking standardized mechanical criteria to ensure consistent quality. Previous efforts to quantify graft properties through uniaxial tensile testing have proven informative but require cutting the tissue into standardized specimens and are therefore not readily suited to routine quality-control workflows. To address this, we investigated puncture testing as a simpler practical alternative that does not require tissue cutting and assessed its correlation with tensile testing and surgeon-based evaluation. Self-assembled dermal and urethral constructs were evaluated using uniaxial tensile and puncture tests, and results were correlated with the surgeon’s qualitative assessments during graft handling. Our findings reveal a strong linear relationship between two mechanical testing techniques. Puncture testing also showed a strong relationship with surgeon-based qualitative assessments of graft handling. This study highlights the potential of puncture testing as a practical mechanical assessment tool for characterizing graft mechanical behavior in the context of surgical handling, and offers a pathway toward the development of standardized, objective quality control protocols for tissue-engineered urethral grafts prior to implantation.

## 1. Introduction

Urethral reconstruction is required for the treatment of a variety of congenital and acquired conditions, including hypospadias, strictures, trauma, and iatrogenic injuries. These conditions can significantly impair urinary and sexual function, as well as the patient’s quality of life and, therefore, often require surgical intervention to restore both anatomy and function [1,2].

Several reconstructive strategies are currently available, including the use of buccal mucosa grafts and natural or synthetic biomaterials, either acellular or cell-seeded. However, these approaches remain limited by donor tissue availability, donor-site morbidity, graft failure, and postoperative complications [3,4,5]. Tissue engineering has therefore emerged as a promising alternative for autologous therapies, particularly through the self-assembly method, which relies exclusively on stromal cells to generate biologically relevant constructs without the use of exogenous biomaterials [6]. These self-assembled tissues have shown histological and mechanical properties comparable to native urethral tissue and thus have a good potential for clinical translation [7]. Of note, self-assembled skin substitutes showed a structure and functionality very similar to native human skin and are currently used in clinical trials [8].

Despite these advances, a major challenge remains: although general assessment criteria have been proposed for tissue-engineered constructs [9,10], standardized methods for evaluating the mechanical properties and surgical handling characteristics of urethral grafts prior to implantation remain poorly established. Consequently, graft selection remains largely dependent on the surgeon’s intraoperative handling and subjective judgment. This evaluation is inherently variable and lacks standardized criteria, limiting reproducibility and making it difficult to ensure consistent graft quality and associated outcomes. In previous studies, this issue was addressed by using uniaxial tensile testing to quantify mechanical properties of self-assembled tissues prior to implantation [7]. While informative, this method is time-consuming and requires sample cutting in a precise shape.

To overcome these limitations, puncture testing has been explored as a more clinically practical alternative. However, no studies have systematically compared uniaxial tensile and puncture testing in this context, and it remains unknown whether these approaches provide equivalent mechanical information. In addition, no validated threshold values currently exist to define whether an engineered tissue is suitable for suturing and grafting, preventing the implementation of objective pre-implantation quality control evaluation.

In this study, we addressed these gaps by performing both uniaxial tensile and puncture testing on self-assembled dermal and urethral constructs, including stromal and epithelialized constructs. Mechanical parameters obtained from both methods were compared and correlated with the surgeon’s qualitative assessment during tissue handling. The objectives were to determine which testing method best reflects surgeon-perceived graft quality and to establish empirical mechanical thresholds for preimplantation assessment to guide tissue selection and improve standardization of engineered graft production.

## 2. Materials and Methods

### 2.1. Ethics Statement

This study was conducted according to the Declaration of Helsinki and was approved by the institution’s committee for the protection of human participants (Comité d’éthique de la recherche du CHU de Québec-Université Laval, protocol number 2012-1341). All patients provided informed written consent prior to conducted biopsies.

### 2.2. Cell Isolation and Culture

Urethral fibroblasts (UF) and urethral urothelial cells (UUC) were isolated from human urethral biopsies according to a recently developed protocol [11]. Briefly, the stroma was separated from the urothelium following overnight incubation at 4 °C in Dulbecco-Vogt modified Eagle’s medium (DMEM) low glucose without phenol (Sigma-Aldrich, Saint Louis, MO, United States) supplemented with 20 mM HEPES, 0.15% fatty acid-free bovine serum albumin (BSA; Proliant, Ankeny, IA, USA), and 2.5 U/mL dispase II (Roche Diagnostics, Mannheim, Germany), followed by an additional 1 h incubation at 37 °C. Urethral fibroblasts were enzymatically dissociated from the stroma using 2.5 U/mL collagenase I/II (CustomBiotech, Penzberg, Germany) and 5 U/mL elastase (Serva, Heidelberg, Germany) for 4 h at 37 °C under gentle agitation and cultured in DMEM supplemented with 10% fetal bovine serum (FBS; Hyclone, Logan, UT, USA), 100 U/mL penicillin, and 25 μg/mL gentamicin (Fisher Scientific, Ottawa, ON, Canada) (fibroblast medium). The urothelium was mechanically separated from the stroma using fine forceps, dissociated by incubation in 0.05% trypsin/EDTA for 30 min at 37 °C under gentle agitation, and the resulting UUC were cultured in a supplemented 3:1 mixture of DMEM and Ham’s F12 medium (DH medium). The DH medium was supplemented with 5% FBS, 0.4 μg/mL hydrocortisone (Teva Canada Ltd., Scarborough, ON, Canada), 5 μg/mL insulin (Sigma Aldrich, St. Louis, MO, USA), 10 ng/mL epidermal growth factor (EGF) (Austral Biologicals, San Ramon, CA, USA), and 0.212 μg/mL isoproterenol hydrochloride (Sandoz Canada, Boucherville, QC, Canada). Urethral biopsies were obtained from transgender female donors undergoing gender-affirming surgery (22, 32, 38, 51, 72, and 77 years old; Table 1).

Dermal fibroblasts (DF) and keratinocytes (K) were isolated from human skin biopsies as previously described [12]. Briefly, the stroma was separated from the epidermis by overnight incubation at 4 °C in 500 μg/mL thermolysin (Sigma Chemicals, St. Louis, MO, USA). Dermal fibroblasts were enzymatically dissociated from the stroma using collagenase H and cultured in DMEM supplemented with 10% FBS, 100 U/mL penicillin, and 25 μg/mL gentamicin. Keratinocytes were isolated from the separated epidermis by trypsin digestion for 30 min at 37 °C and cultured in DH medium. Dermal biopsies were obtained from female donors aged 25, 34, 36, 38, 51, 54, and 56 years (Table 1). All cells were cryopreserved until use and were used at passages 2–4.

### 2.3. Reconstruction of 3D Flat Urethral and Dermal Constructs

**Figure 1:**
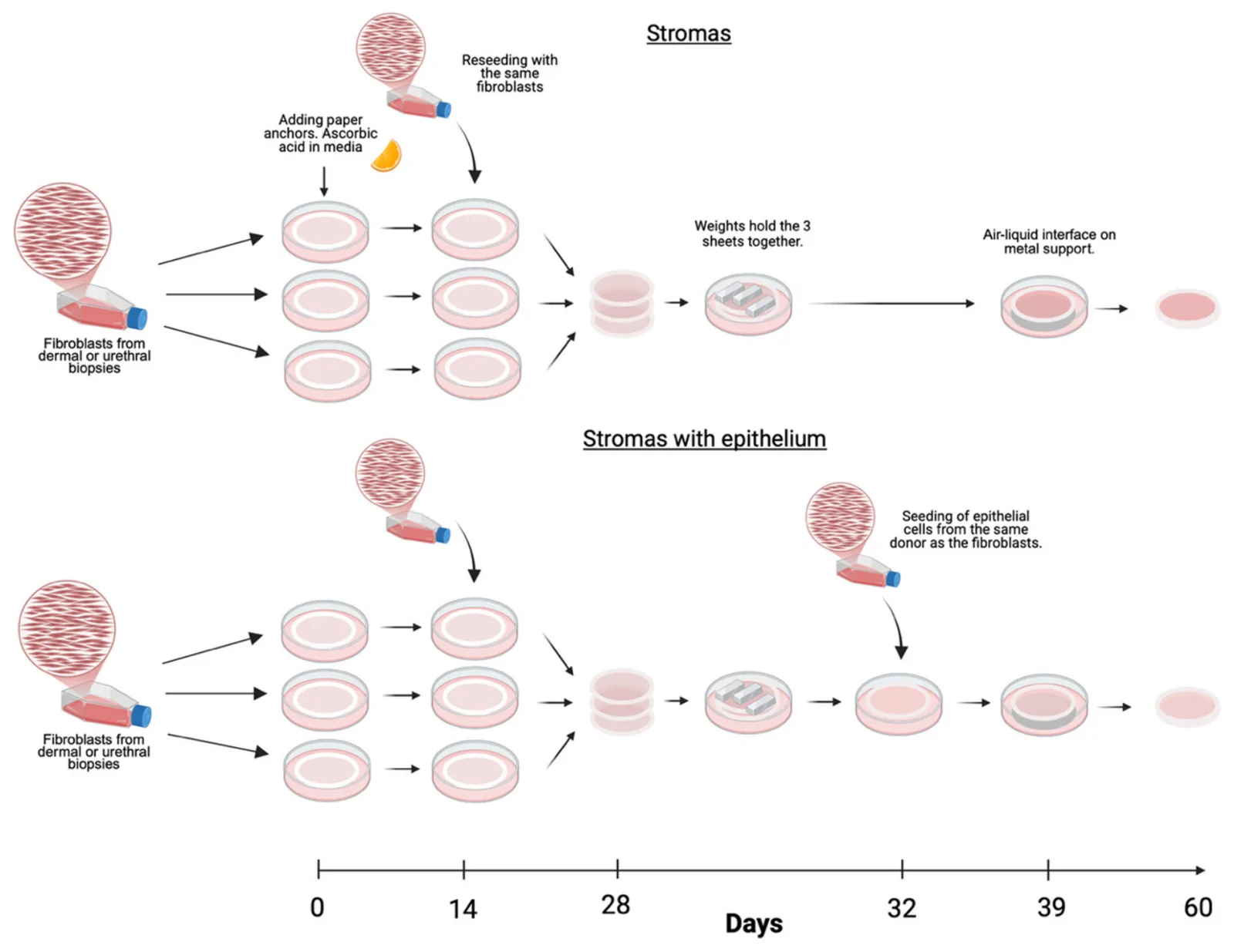
Production of the tissue-engineered constructs by the self-assembly method.

The self-assembly approach was used to produce tridimensional reconstructed tissues, according to standard methods previously described [13]. Briefly, the fibroblasts were seeded to obtain a final concentration of 5 × 10^4^ cells/cm^2^ in 6-well plates containing an anchoring ring (Falcon, Durham, NC, USA) and cultured in the same fibroblast medium supplemented with 50 µg/mL ascorbate (Figure 1). A second cell seeding was performed on day 14 at the same initial density on the newly formed cellularized stromal sheets. The culture was pursued until enough newly synthesized extracellular matrix (ECM) had accumulated to form a sheet, which commonly corresponded to 28 days. Next, three stromal sheets were superimposed and were maintained in culture for 4 additional weeks. For epithelialized constructs, 5 × 10^4^ epithelial cells were seeded onto each construct a few days after stromal sheets superimposition. Four days later, 32 days after the initial seeding, epithelialized tissues were elevated to the air-liquid interface for 3 weeks to induce epithelial differentiation. Stromal-only tissues were subjected to the same air-liquid interface culture period to maintain identical maturation conditions and allow direct comparison between epithelialized and non-epithelialized constructs. This approach was applied to all constructs throughout the study to preserve consistency with the self-assembly maturation protocol and to avoid introducing additional culture-related variables during mechanical characterization. Biological replicates were defined as independent donor populations, with N = 7 DF and N = 6 UF donor populations included in this study. Multiple reconstructed tissues were generated independently from each donor population and were considered reconstructed tissue replicates (n = 8–12 reconstructed tissue replicates per donor population). These tissue replicates were generated to account for the inherent variability associated with the self-assembly approach and to provide a representative assessment of the properties of tissues derived from each donor. Donor-to-donor variability was considered at the biological replicate level, whereas variability among tissues generated from the same donor was assessed at the reconstructed tissue replicate level.

### 2.4. Tissue Thickness Measurement by Non-Contact Optical Micrometer

**Figure 2:**
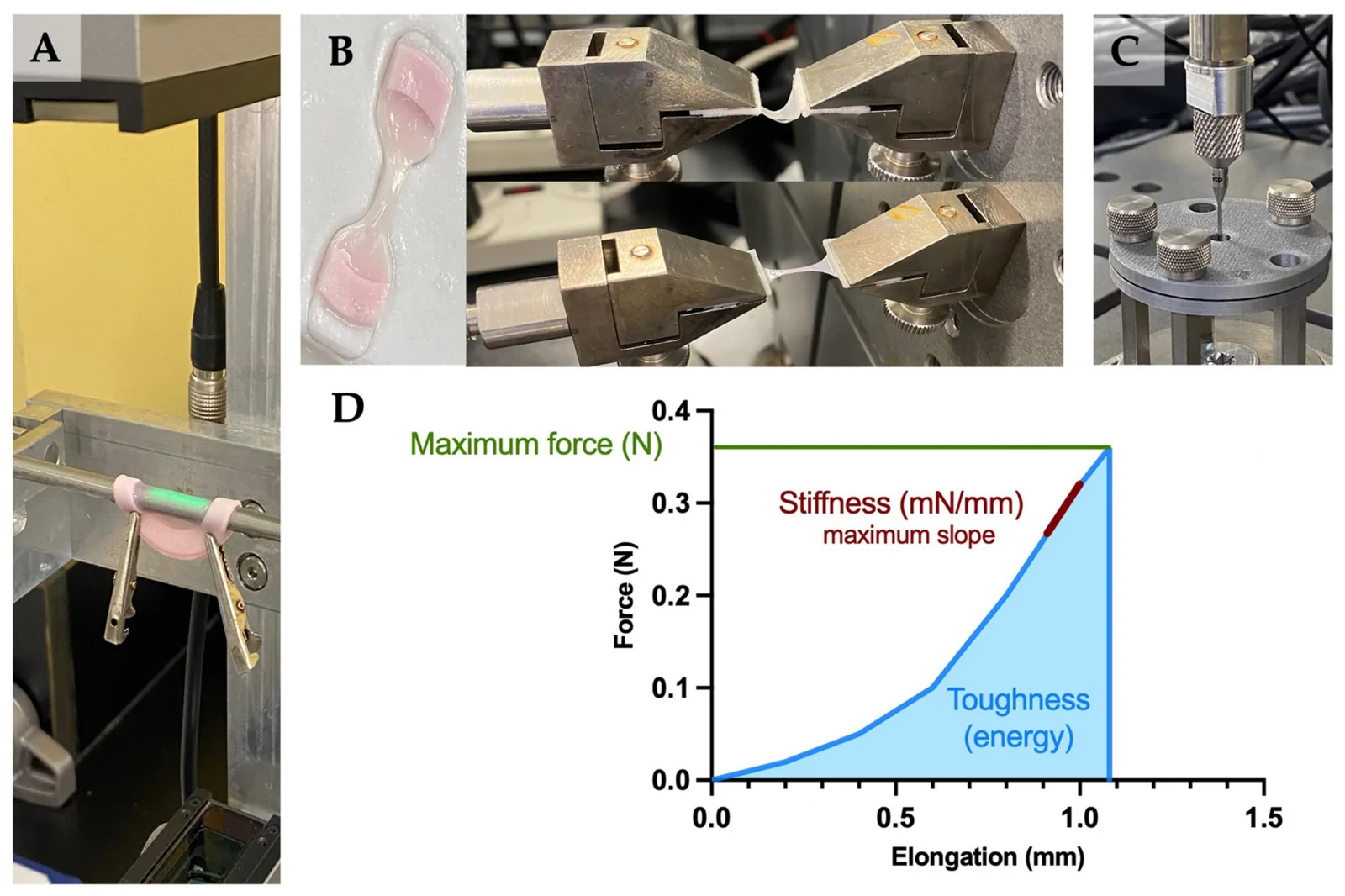
Mechanical testing. (**A**) Thickness measurement using the Keyence laser. (**B**) Uniaxial tensile testing using the Electropuls E1000. (**C**) Puncture testing using the Electropuls E1000. (**D**) Representative curve used to measure the different mechanical parameters obtained from both tests.

Immediately before mechanical testing, the thickness of each stromal construct was measured using a non-contact optical micrometer with a precision of ±2 μm (LS-760, Keyence, Osaka, Japan). This laser-based system determines thickness by measuring the shadow cast by the sample as it interrupts the light beam between the emitter and the receiver. Constructs remained attached to their paper anchoring rings and were folded over a small metal bar (4.76 mm diameter) and clasped at the bottom without interfering with the light beam (Figure 2). The thickness of the metal bar was first used as the zero reference, and because the tissue was folded around the bar, the measured value was divided by two to obtain the thickness of a single tissue layer. Three measurements were obtained for each construct, and the mean value was used for analyses.

### 2.5. Mechanical Testing

Mechanical properties of the flat constructs were assessed by uniaxial tensile testing and puncture testing using an ElectroPuls E1000 mechanical tester with a 10 N loading cell (sensitivity of 0.0005 N) (Instron, Norwood, MA, USA). For each testing method, the order in which the constructs were tested was randomized to minimize potential bias related to testing sequence.

#### 2.5.1. Uniaxial Testing

For uniaxial tensile testing, one dog-bone-shaped specimen was obtained from each tissue construct using a custom-made stainless-steel punch (total length: 36 mm; gauge length: 10 mm; gauge width: 2 mm; end widths: 11 mm). The specimen was clamped at both ends, and one clamp was displaced at a constant rate of 0.2 mm/s until tissue rupture. For each donor, at least four stromal constructs and four epithelialized constructs were tested. Force and displacement were recorded to assess the tissue’s mechanical resistance. Strain (force by elongation) curves were generated. All data were expressed as means and standard deviations, and the graphs were generated using GraphPad Prism v.9.2 Software (San Diego, CA, USA). Maximum force (N) was defined as the peak force reached immediately prior to sample rupture. Stiffness (mN/mm) was determined as the slope in the linear section of the j-shaped curve. Toughness (energy) was calculated as the area under the force–displacement curve from the start of the test to the rupture of the sample, representing the total energy absorbed by the material before failure.

#### 2.5.2. Puncture Testing

Puncture testing was performed according to Rioux et al., 2026 [14]. The whole construct was placed flat at the center of a puncture fixture with a 5 mm diameter aperture. A spherical probe (2 mm diameter) mounted on a needle was used on the Instron ElectroPuls E1000 machine (Norwood, MA, USA) and was positioned directly above the center of the tissue. A  ±  10 N capacity load cell with an accuracy of  ± 2.5 mN was used. During the test, the needle moved vertically at a constant rate of 0.02 mm/s to puncture the tissue through the opening. Four constructs per donor (four stromas and four stromas with epithelium) were tested. Force and displacement were recorded to assess the tissue’s mechanical resistance. Strain (force by elongation) curves were generated. All data were expressed as means and standard deviations, and the graphs were generated using GraphPad Prism v.9.2 Software (San Diego, CA, USA). Maximum force, stiffness and toughness were measured as described above.

### 2.6. Surgeon-Based Handling Test

An experienced surgeon performed a manual handling assessment on stromal tissues (n ≥ 3 per population) with an independent non-surgeon observer present to document and verify the surgeon’s assessment. The surgeon was blinded to donor identity, tissue origin, expected construct quality, and mechanical test results, and the constructs were presented in randomized order. Each construct was incised centrally (1.2–1.5 cm) using a scalpel to reproduce the type of tissue manipulation encountered during surgical handling. Two to three sutures were then placed along the incision using Vicryl 6-0 threads (Ethicon, North Ryde, NSW, Australia). The surgeon then gently pulled on two opposing sides of the tissue to evaluate its handling during and after suturing. The assessment focused on the ease of tissue manipulation, the ability of the tissue to hold the sutures, its resistance to deformation and tearing at the suture sites, and its overall response to the applied tension. Based on these characteristics, the surgeon assigned a qualitative score from 0 to 4, with 0 indicating the weakest tissue response and 4 indicating the strongest tissue response during handling and suturing. This qualitative assessment was selected to evaluate tissue behavior during manipulation and suturing under conditions that more closely reflect the surgical context.

### 2.7. Statistics

Statistical analyses were performed using GraphPad Prism v.9.2 software (San Diego, CA, USA). Results are expressed as means ± standard deviations. A two-tailed paired *t*-test was used to compare epithelialized and non-epithelialized constructs from the same fibroblast populations. Simple linear regression was used to assess the linear relationships between mechanical properties measured by tensile and puncture testing. The coefficient of determination (R^2^) was used to quantify the strength of these linear relationships. Statistical significance was indicated as follows: ns: not significant, *p* > 0.05; * *p* ≤ 0.05; ** *p* ≤ 0.01; *** *p* ≤ 0.001; **** *p* ≤ 0.0001. To assess the relationship between mechanical testing results and surgeon evaluations, an interpretation framework was established by dividing the plots into four quadrants: true positive, when mechanical testing indicated adequate tissue properties and the surgeon rated the tissue as satisfactory; true negative, when mechanical testing indicated poor tissue properties and the surgeon rated the tissue as unsatisfactory; false positive, when mechanical testing indicated adequate tissue properties but the surgeon rated the tissue as unsatisfactory; and false negative, when mechanical testing indicated poor tissue properties but the surgeon rated the tissue as satisfactory. A minimum of 4 reconstructed tissue replicates per condition was targeted to obtain a representative assessment of tissue properties given the inter-donor variability observed with the self-assembly approach. More than 4 tissues were initially generated to account for potential tissue loss during the reconstruction process. When more than 4 tissues successfully reached the final stage, all available tissues were included in the mechanical testing.

## 3. Results

Comparison of the uniaxial tensile test and the puncture test

**Figure 3:**
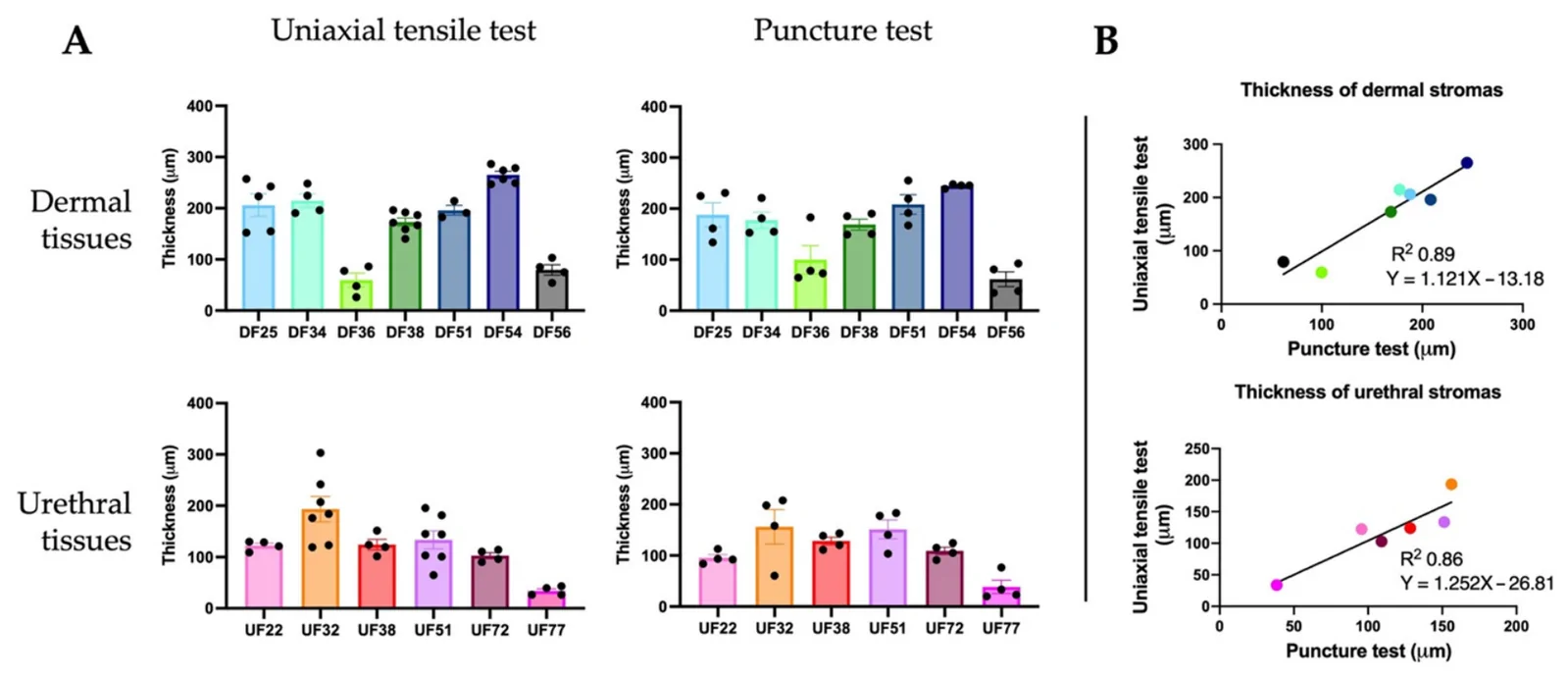
Stromal thickness of the constructs. (**A**) Mean thicknesses (µm) of the different dermal (DF) and urethral (UF) stromal populations used for uniaxial tensile testing and puncture testing. Each column represents a stromal population ranked according to donor age. Each dot represents an independent construct, considered a biological replicate. (**B**) Correlation between the mean thicknesses obtained from both tests for dermal and urethral stromas. Color code is the same as that used in panel A.

After two months of culture, reconstructed stromal tissues had matured enough to be suitable for analysis. Four samples per population were subjected to uniaxial tensile testing and four to puncture testing. Stromal thickness was measured by laser prior to mechanical testing. Thickness varied across donors and did not correlate with age (Figure 3A). Thickness measurements obtained for samples undergoing uniaxial tensile testing and puncture testing were comparable, with coefficients of determination (R^2^) of 0.89 for dermal constructs and 0.86 for urethral constructs (Figure 3B). These strong associations indicate that tissue samples were comparable between the two testing groups and that thickness was homogeneous, which supports overall uniformity across the experimental conditions.

**Figure 4:**
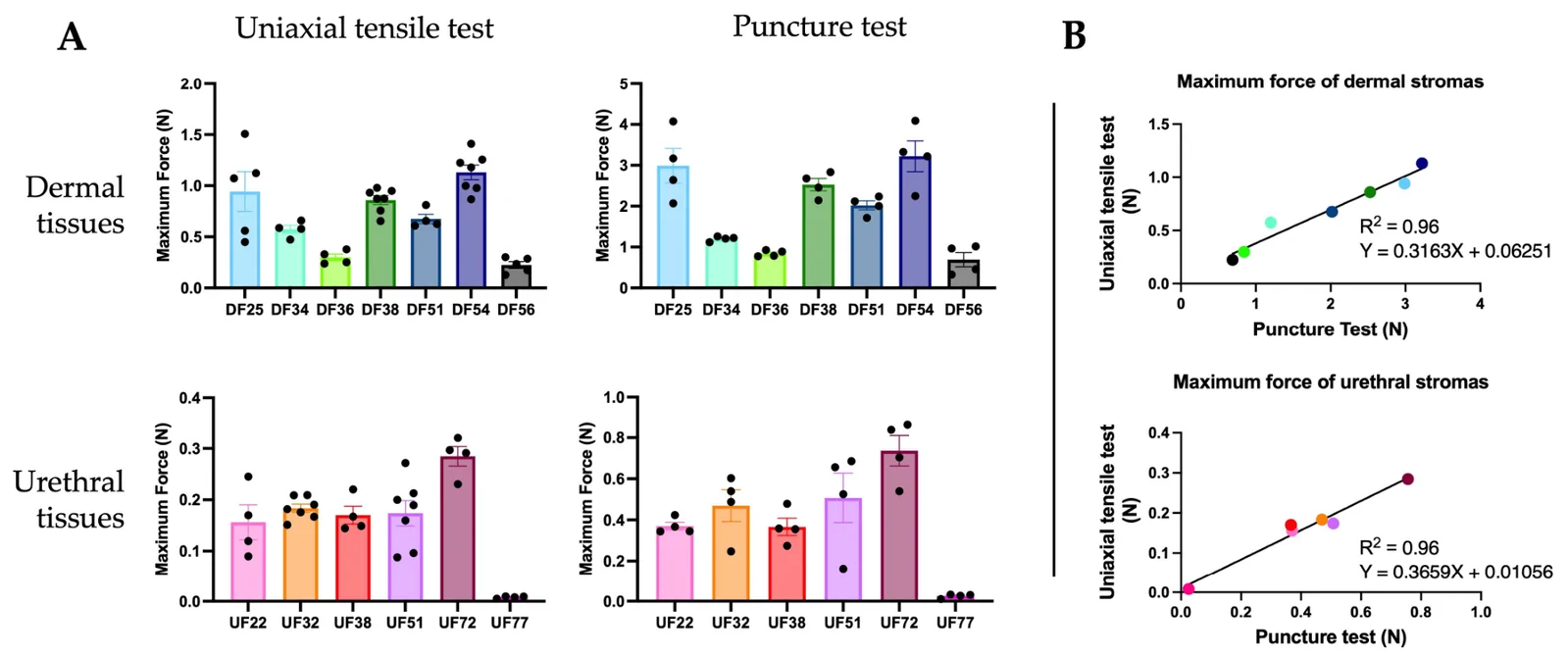
Maximum force of the constructs. (**A**) Mean maximum forces in Newtons (N) of the different dermal (DF) and urethral (UF) stromal populations used for uniaxial tensile testing and puncture testing. Each column represents a stromal population ranked according to donor age. Each dot represents an independent construct, considered a biological replicate. (**B**) Correlation between the mean maximum forces obtained from both tests for dermal and urethral stromas. Each dot represents the mean of the values of a stromal population. Color code is the same as that used in panel A.

The first mechanical parameter evaluated was maximum force (N), defined as the highest force measured prior to the sudden failure of the sample. Maximum force values also exhibited inter-donor variability (Figure 4A). However, when comparing values obtained from uniaxial tensile testing and puncture testing, a strong association was observed, with R^2^ values of 0.96 for both dermal and urethral stromal tissues. This indicates a strong association between the two testing methods for these constructs in this specific protocol, with similar slopes (0.3163 for dermal tissues and 0.3659 for urethral tissues) (Figure 4B).

**Figure 5:**
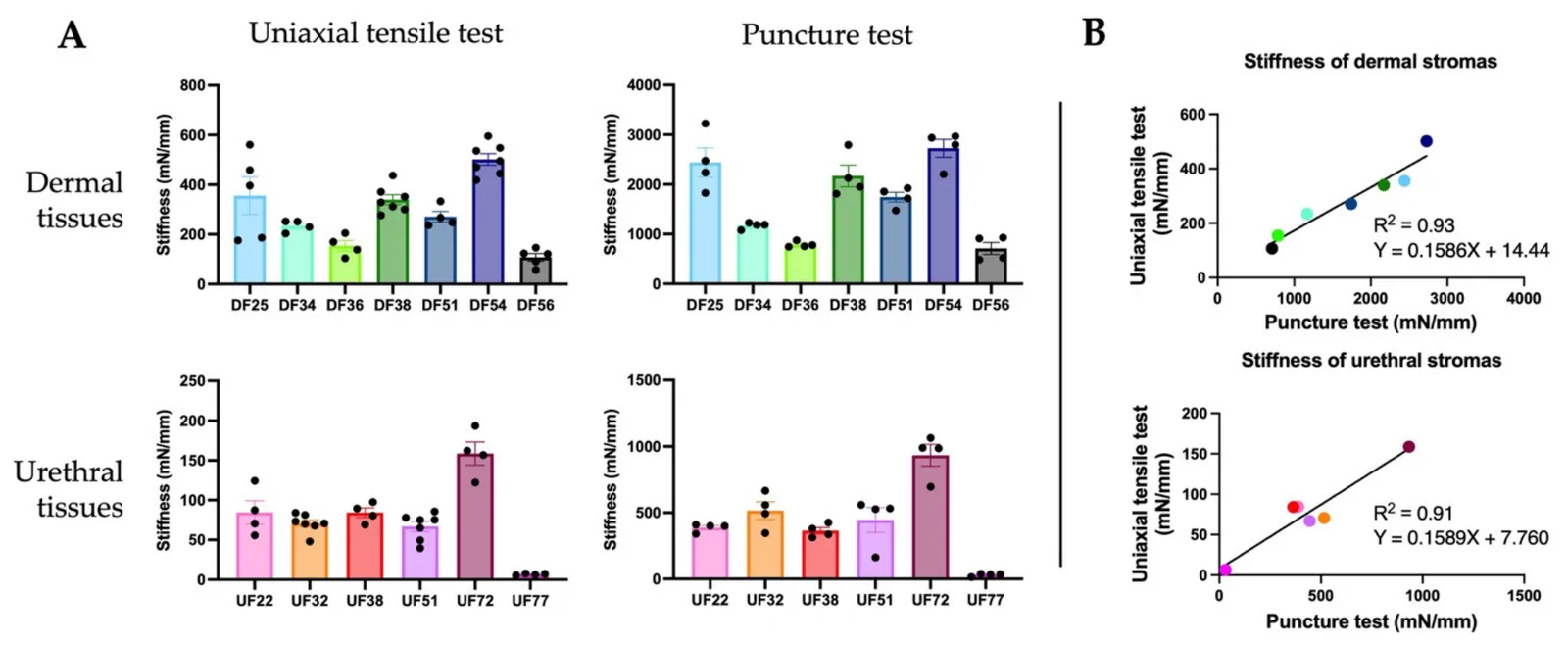
Stiffness of the constructs. (**A**) Mean stiffness values (mN/mm) of the different dermal (DF) and urethral (UF) stromal populations measured by uniaxial tensile testing and puncture testing. Each column represents a stromal population ranked according to donor age. Each dot represents an independent construct, considered a biological replicate. (**B**) Correlation between the mean stiffness values obtained from both tests for dermal and urethral stromas. Color code is the same as that used in panel A.

The second parameter measured was stiffness (mN/mm). This parameter reflects the resistance of a tissue to deformation under an applied force. Similar trends were observed for stromal stiffness. While substantial stiffness variability was noted between donors (Figure 5A), a strong association was found between uniaxial tensile and puncture test results, with R^2^ values of 0.93 and 0.91 for dermal and urethral stromal tissues, respectively (Figure 5B). Again, comparable slopes were obtained for both tissue types (0.1586 for dermal tissues and 0.1589 for urethral stromal tissues).

**Figure 6:**
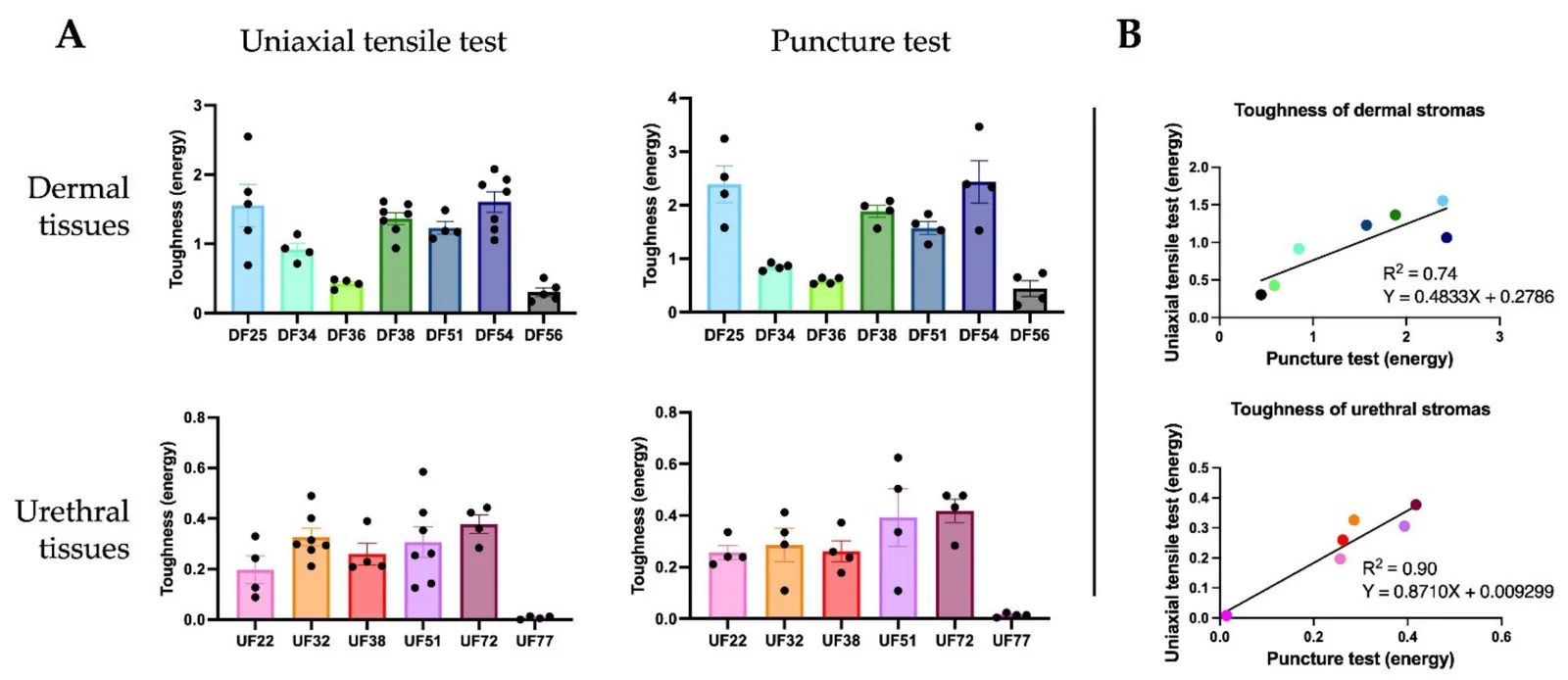
Toughness of the constructs. (**A**) Mean toughness values (energy) of the different dermal (DF) and urethral (UF) stromal populations used for uniaxial tensile testing and puncture testing. Each column represents a stromal population ranked according to donor age. Each dot represents an independent construct, considered a biological replicate. (**B**) Correlation between the mean toughness values obtained from both tests for dermal and urethral stromas. Color code is the same as that used in panel A.

The third parameter assessed was toughness (energy). It represents the total energy required to initiate sample rupture. For toughness, although similar overall trends were observed (Figure 6A), discrepancies between the two testing methods were more pronounced for dermal tissues (R^2^ = 0.74), whereas a stronger association was maintained for urethral stromal tissues (R^2^ = 0.90) (Figure 6B).

Overall, the mean coefficient of determination across the mechanical properties and both fibroblast-derived tissue types was around 0.90, indicating a high level of correlation between the two testing methods. In addition, the mean coefficients of variation calculated for the different mechanical properties in both dermal and urethral tissues were generally lower with puncture testing than with uniaxial tensile testing (Table 2), indicating reduced variability for most parameters with the puncture approach.

Mechanical Properties of Epithelialized and Non-Epithelialized Stromas Assessed by Puncture Testing

Before performing the surgeon’s suture assessment, it was necessary to determine whether epithelialization influences the mechanical properties of the engineered tissues. In clinical applications, surgeons would manipulate epithelialized grafts; however, the production of epithelialized constructs involves additional cell seeding procedures and requires increased resources and costs compared with non-epithelialized constructs. Therefore, if no significant mechanical differences are observed between epithelialized and non-epithelialized constructs, surgeon evaluation and mechanical assessments could be performed using non-epithelialized constructs prior to implantation, as these constructs are simpler and less expensive to produce and most of the mechanical properties are provided by the stromal compartment.

Since the uniaxial tensile and puncture tests showed strong correlation for mechanical properties of the self-assembled dermal and urethral constructs evaluated in this study, puncture testing was selected for this comparison because it is quicker to perform and involves localized contact forces more representative of surgical suturing. The mechanical properties of epithelialized and non-epithelialized dermal and urethral constructs were therefore evaluated by puncture testing using six stromal populations, including three dermal (DF34, DF36 and DF51) and three urethral populations (UF22, UF38 and UF72). The corresponding Masson’s trichrome histological images are presented in Supplementary Figure S7. Detailed plots of the individual mechanical properties are provided in Supplementary Figures S1–S6, while the corresponding raw data are presented in Supplementary Table S1.

**Figure 7:**
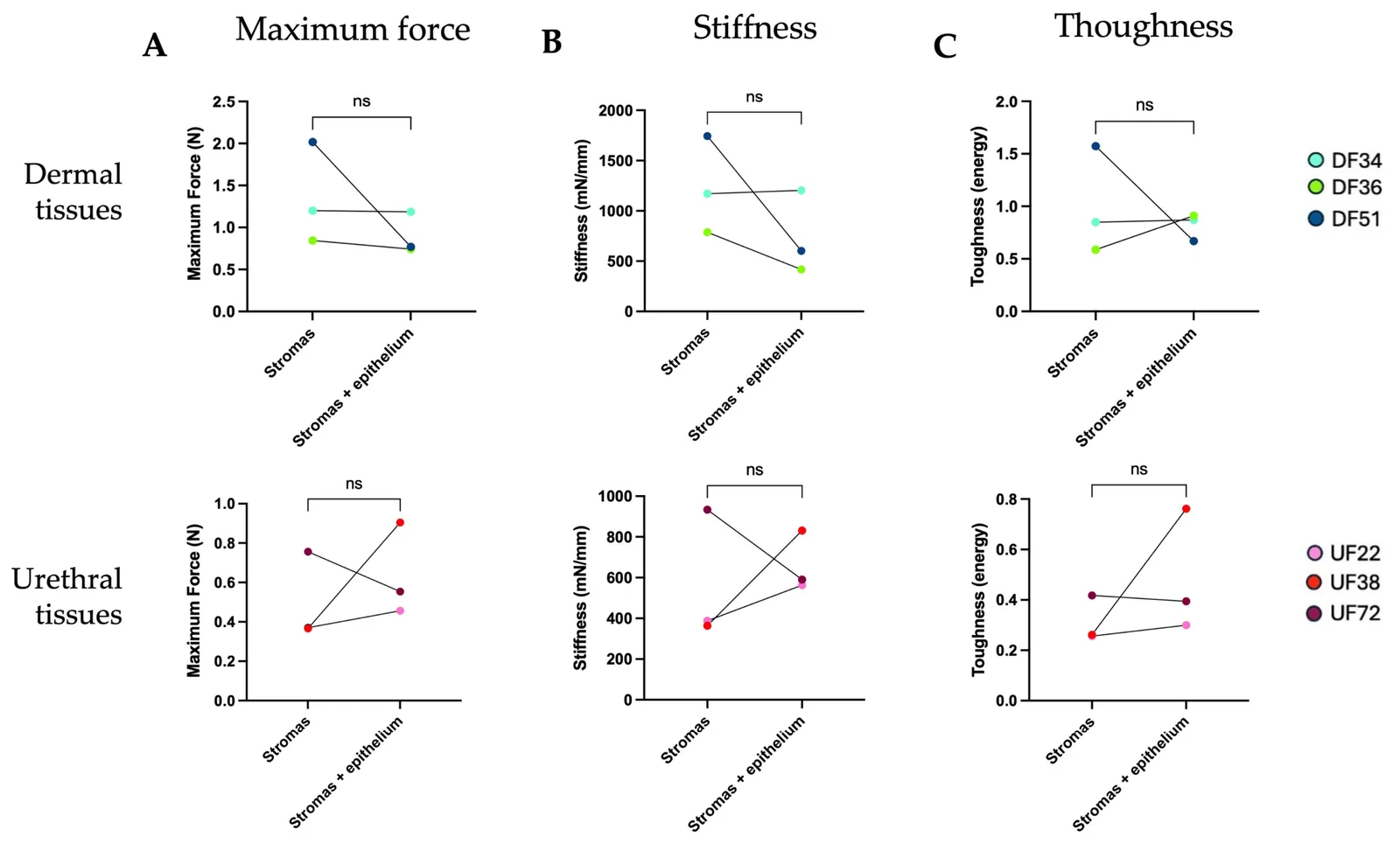
Comparison of mechanical properties obtained by puncture testing for epithelialized and non-epithelialized dermal (DF) and urethral (UF) constructs. Each dot (N = 6 donor populations) represents the mean value of a specific parameter for an individual population (n = 4 reconstructed tissue replicates/donor population). Standard deviations were omitted to clarify the graph. (**A**) Maximum force (N). (**B**) Stiffness (mN/mm). (**C**) Toughness (energy). Statistical analysis was performed using a two-tailed paired *t*-test. Statistical significance was indicated as follows: ns: not significant.

As shown in Figure 7, no significant differences were observed between stromal and epithelialized tissues for any of the mechanical properties evaluated. For urethral fibroblast-derived tissues, maximum force did not differ significantly between conditions (mean difference = 0.14 N, 95% CI: −0.79 to 1.07, *p* = 0.582), nor did stiffness (mean difference = 100.0 mN/mm, 95% CI: −919.8 to 1120, *p* = 0.714) or toughness (mean difference = 0.17 J, 95% CI: −0.53 to 0.88, *p* = 0.402). Similarly, for dermal fibroblast-derived tissues, no significant differences were observed for maximum force (mean difference = −0.46 N, 95% CI: −2.17 to 1.26, *p* = 0.371), stiffness (mean difference = −492.4 mN/mm, 95% CI: −1975 to 990.3, *p* = 0.289), or toughness (mean difference = −0.19 J, 95% CI: −1.77 to 1.40, *p* = 0.665). Each value represents the mean of four tissue constructs generated from cells obtained from the same donor, and statistical comparisons were performed using two-tailed paired *t*-tests. These results indicate that epithelialization does not significantly alter stromal mechanical properties at a scale that will affect the ability of the surgeon to handle the tissue. Consequently, non-epithelialized constructs were used for the subsequent surgeon’s suture assessment and for the analysis of correlations between the surgeon’s evaluation and tissue mechanical properties.

Investigating relationship between mechanical properties and the surgeon’s evaluation

**Figure 8:**
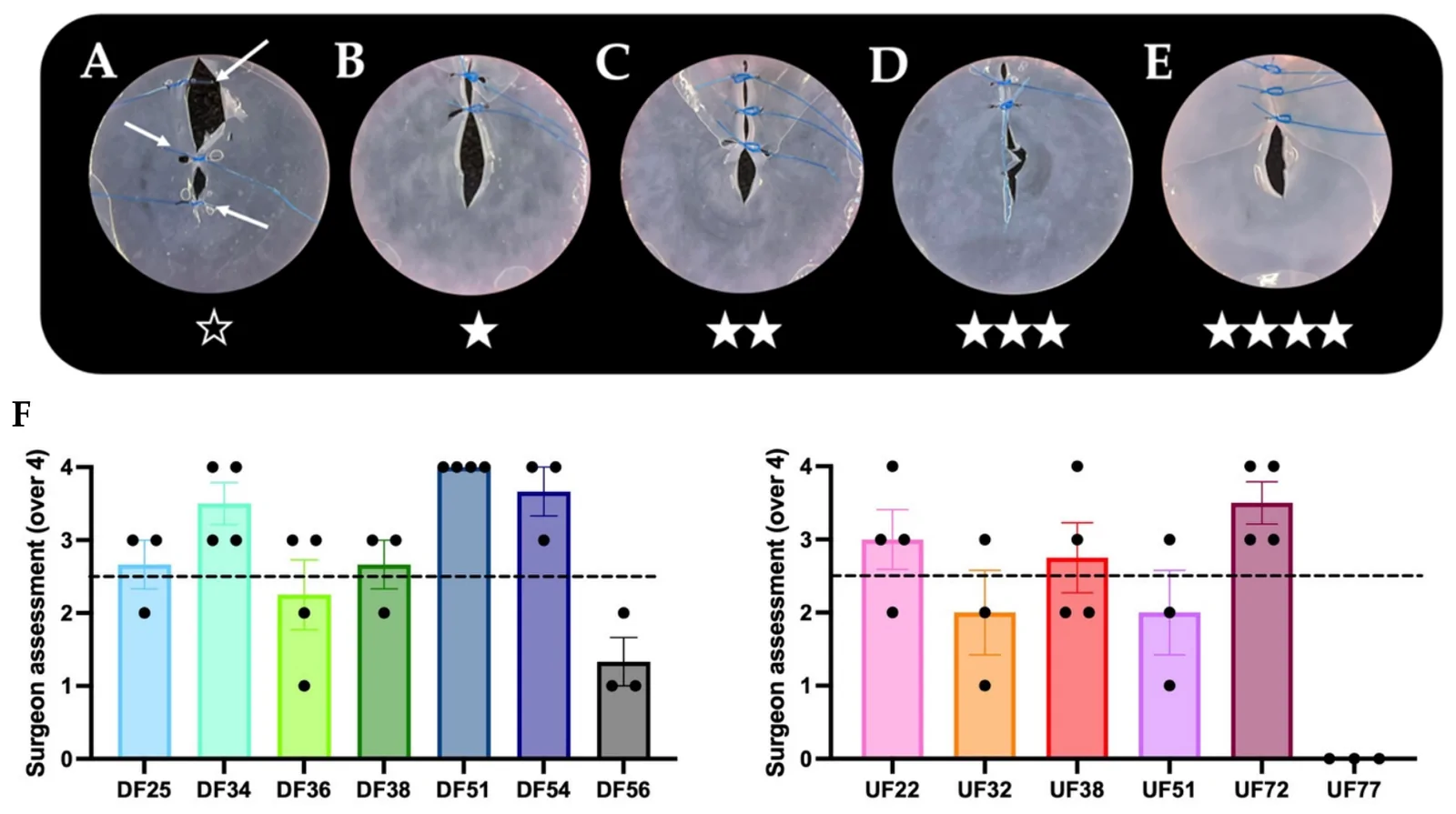
The surgeon’s qualitative assessment during graft handling. (**A**) Grade 0 (✩): immediate tearing upon incision. (**B**) Grade 1 (★): Non-suturable. (**C**) Grade 2 (★★): Suturable with extensive tears. (**D**) Grade 3 (★★★): Suturable with minimal tearing. (**E**) Grade 4 (★★★★): Suturable without tears. (**F**) Each column represents the mean grade of stromal constructs from one fibroblast population (n = 3–4 reconstructed tissue replicates). The dotted bars represent the mean +/− the standard deviation of the means of the constructs. Dermal (DF) and urethral (UF) constructs.

As illustrated in Figure 8A–F, after handling the tissue (incision and sutures), the DF51 population was the most favored by the surgeon, with a mean score of 4 out of 4. This was followed by DF54 and DF34, which achieved a mean score of 3.7 and 3.5, respectively. The urethral population UF72 exhibited the highest mean performance of the UF group, with a mean score of 3.5. The dermal population DF56 exhibited a lower mean score of 1.33, while UF77 showed the lowest performance, with a mean score of 0.

As shown in the previous results, a strong correlation was observed between the mechanical properties measured by the two testing methods (especially for maximum force and stiffness), indicating that both methods provide concordant assessments of the mechanical performance of our engineered tissues produced and assessed under these conditions. Given the practical advantages of puncture testing, puncture-derived mechanical parameters were selected for correlation with the surgeon’s qualitative assessment during suturing.

**Figure 9:**
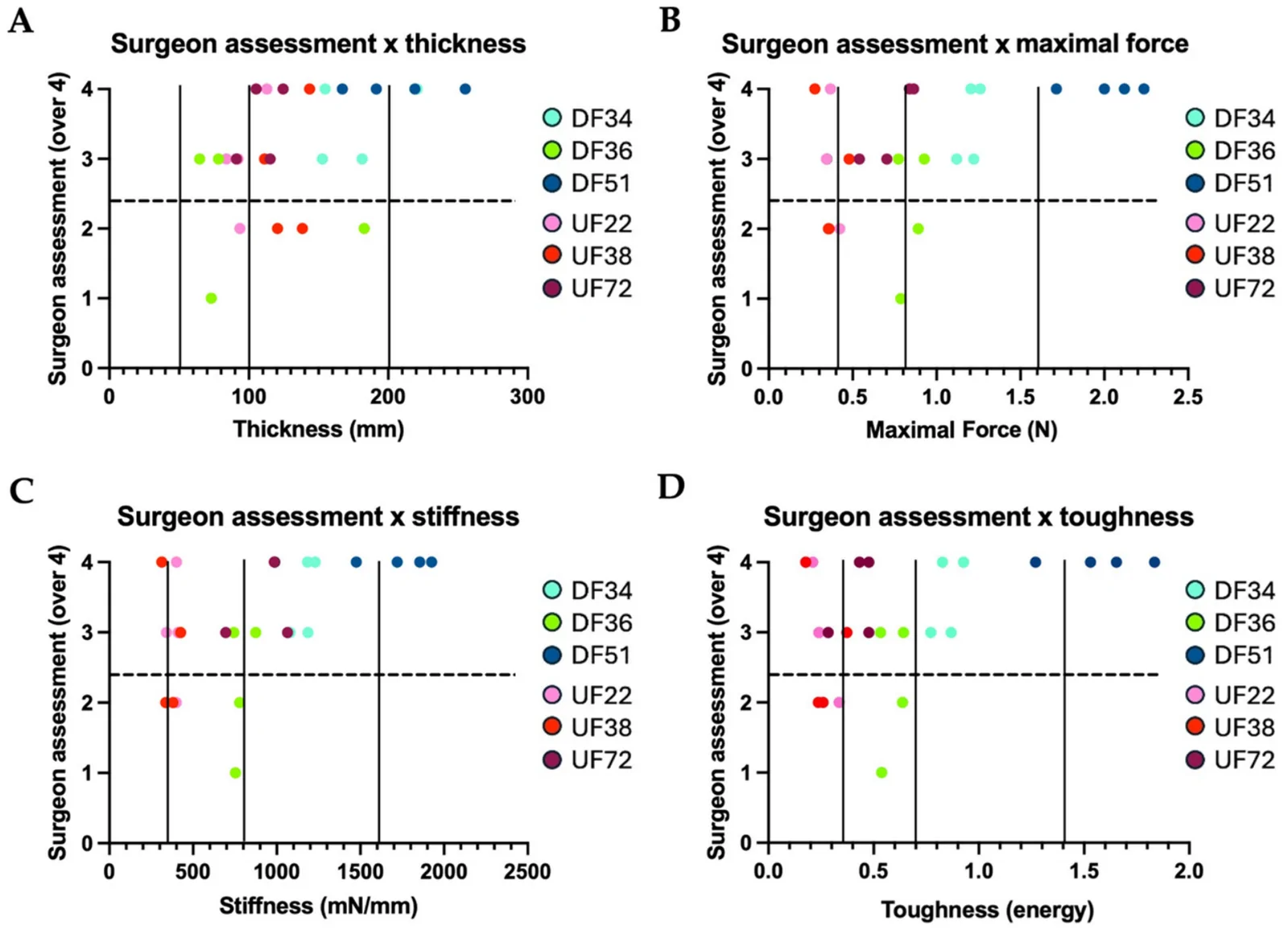
Correlations between mechanical properties and the surgeon’s evaluation of dermal and urethral stromas. Each dot represents the value obtained for a single sample of urethral (UF) or dermal (DF) stroma. The dashed line represents the threshold corresponding to a score of 2.5 for suturing. Vertical lines represent threshold values used for score assignment. (**A**) Thickness. (**B**) Maximum force. (**C**) Stiffness. (**D**) Toughness.

The relationships between each mechanical property and the surgeon’s suture handling scores are presented in Figure 9. Each point represents an individual stromal sample from the six tissue populations evaluated (three dermal and three urethral populations), with four stromal samples per population. The x-axis represents the measured value of the mechanical property, while the y-axis represents the score assigned by the surgeon during the suture test. A horizontal threshold line was set at a score of 2.5 to distinguish tissues considered unsuitable (<2.5) from those considered suitable (>2.5) for surgical handling. This cutoff was based on the surgeon’s perception of tissue quality with a score of 2 representing a less favorable assessment, and a score of 3 representing a favorable assessment (Figure 8).

Overall, samples with higher values of the evaluated mechanical properties tended to receive higher surgeon scores and showed improved suture performance. Because direct relationships between ordinal surgeon handling scores and continuous mechanical variables are statistically limited, mechanical outputs were converted into ordinal scores through a calibrated classification system. For each mechanical property, the measured values were plotted against the surgeon handling score. A surgeon score of 2.5 was selected as the threshold separating acceptable (≥2.5) from unacceptable (<2.5) handling performance. The mechanical value corresponding to a surgeon score of 2.5 was used as the reference threshold for that parameter (Table 3). These thresholds are intended as empirical/exploratory classification cutoffs for relating mechanical properties to surgeon-perceived tissue quality. The values of the mechanical properties, corresponding scores assigned to each stromal sample and the surgeon assessment are detailed in Table 4. For each sample, the measured values of thickness, maximum force, stiffness, and toughness were converted into ordinal scores according to the predefined ranges established in Table 3. To generate a simple and symmetrical four-level classification system around the reference threshold, the lower boundary was defined as one-half of the threshold value and the upper boundary as twice the threshold value, generating four performance classes (very low, low, good, and very good, which were assigned scores from 1 to 4.

The values of the mechanical properties, corresponding scores assigned to each stromal sample and the surgeon’s assessment are detailed in Table 4. For each sample, the measured values of thickness, maximum force, stiffness, and toughness were converted into ordinal scores (1–4) according to the predefined ranges established in Table 3.

To determine the relative contribution of each mechanical property to surgical performance, the distribution of samples within the corresponding quadrants of each mechanical-property graph (Figure 9) was analyzed. True positives (upper-right quadrant) corresponded to samples with mechanical properties classified as satisfactory that were also rated as satisfactory by the surgeon, whereas true negatives (lower-left quadrant) corresponded to samples with poor mechanical properties that were also rated as unsatisfactory by the surgeon. In contrast, false positives (lower-right quadrant) represented samples with satisfactory mechanical properties but an unsatisfactory surgeon rating, while false negatives (upper-left quadrant) represented samples with poor mechanical properties despite a satisfactory surgeon rating. Samples located in the upper-right quadrant (true positives) and lower-left quadrant (true negatives) were considered concordant with the surgeon’s assessment. The sum of true positive and true negative samples was divided by the total number of samples (n = 24), generating an Adequacy Factor (*AF*) for each mechanical parameter:

$$AF=\frac{TP+TN}{n}$$

where *TP* represents true positives, *TN* represents true negatives, and *n* is the total number of samples.

The Adequacy Factor represents the proportion of samples correctly classified by a given mechanical parameter according to the predefined classification system, including both *TP* and *TN* classifications. In the present analysis, the Adequacy Factor was calculated using the same dataset used to establish the mechanical value ranges and to develop the scoring system.

This Adequacy Factor was then incorporated into a weighted mechanical scoring model to calculate a global mechanical score for each sample. For each construct, the score associated with the value of each mechanical property was multiplied by its corresponding Adequacy Factor, and the weighted scores were summed according to the following equation:

$$\begin{array}{cc} \mathrm{Total}\;\mathrm{Mechanical} & \;\mathrm{Score}\\ & =\left(S_{thickness}\times AF_{thickness}\right)+\left(S_{\max force}\times AF_{\max force}\right)+\left(S_{stiffness}\times AF_{stiffness}\right)\\ & +\left(S_{toughness}\times AF_{toughness}\right) \end{array}$$

where *S* represents the score assigned to each parameter based on the predefined value ranges presented in Table 3.

**Figure 10:**
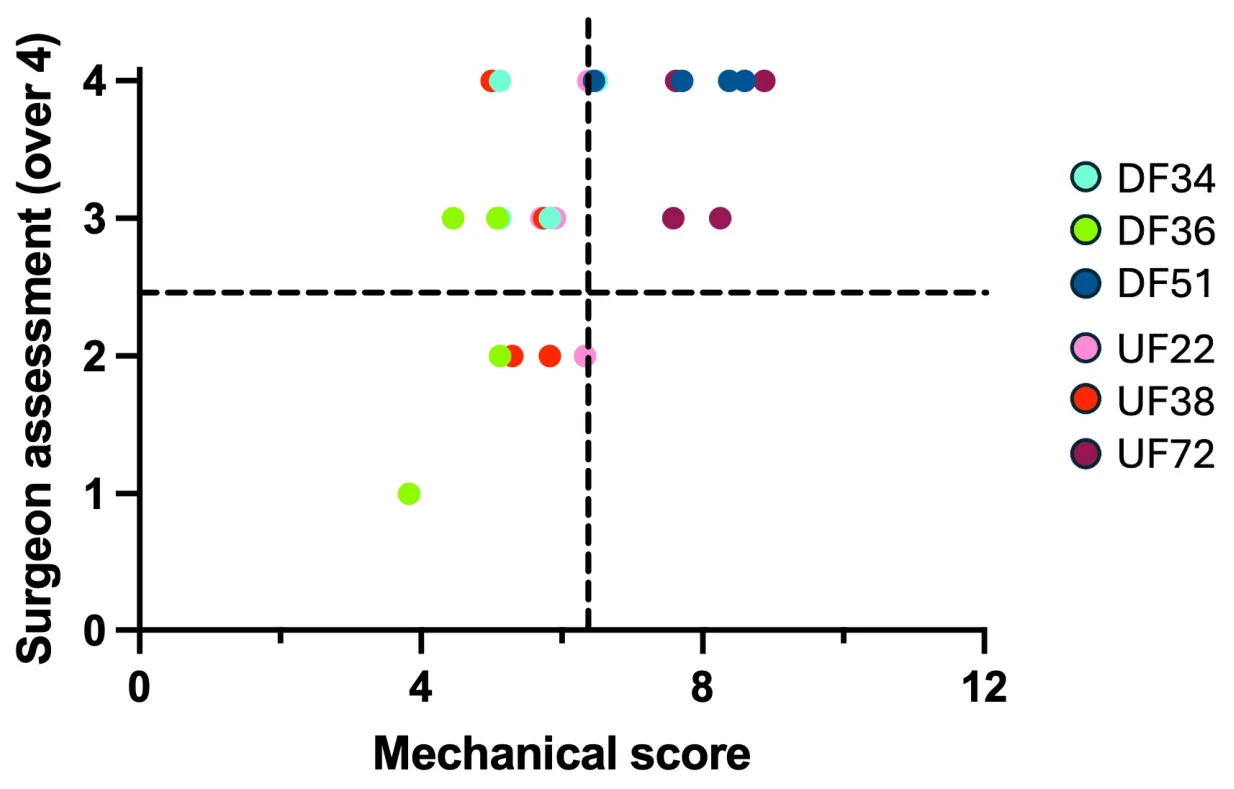
Correlation between the calculated mechanical score and the surgeon’s evaluation of dermal (DF) and urethral (UF) stromas. Each dot represents an individual urethral or dermal stromal sample. The x-axis corresponds to the calculated total mechanical score obtained using the weighted scoring equation, while the y-axis represents the surgeon-assigned score during the suture test. The horizontal dashed line indicates the clinical threshold score of 2.5, used to distinguish tissues considered unsuitable (<2.5) from those considered suitable (>2.5) for surgical handling. The vertical dashed line indicates the corresponding mechanical score threshold (6.35), calculated by applying the weighted scoring equation using an intermediate score value of 2.5 for each mechanical property. These threshold lines divide the graph into four quadrants representing true positives, true negatives, false positives, and false negatives.

The resulting total mechanical scores were compared with the surgeon’s suture handling scores in Figure 10. The graph was divided into four quadrants to evaluate the relationship between mechanical assessment and surgical perception: upper-right (true positives), lower-left (true negatives), lower-right (false positives), and upper-left (false negatives). True positive and true negative samples represented correlation between mechanical characterization and surgical assessment, whereas false positive and false negative samples represented discordant classifications. Among all samples, 41.7% were classified as true positives and 20.8% as true negatives, representing a total correlation rate of 62.5% between mechanical characterization and surgeon evaluation. In contrast, 37.5% of samples were classified as false negatives, while no samples were observed in the false-positive quadrant. Overall, the predominance of true classifications suggests a correlation between mechanical assessment and the surgeon’s perception of suture handling.

**Figure 11:**
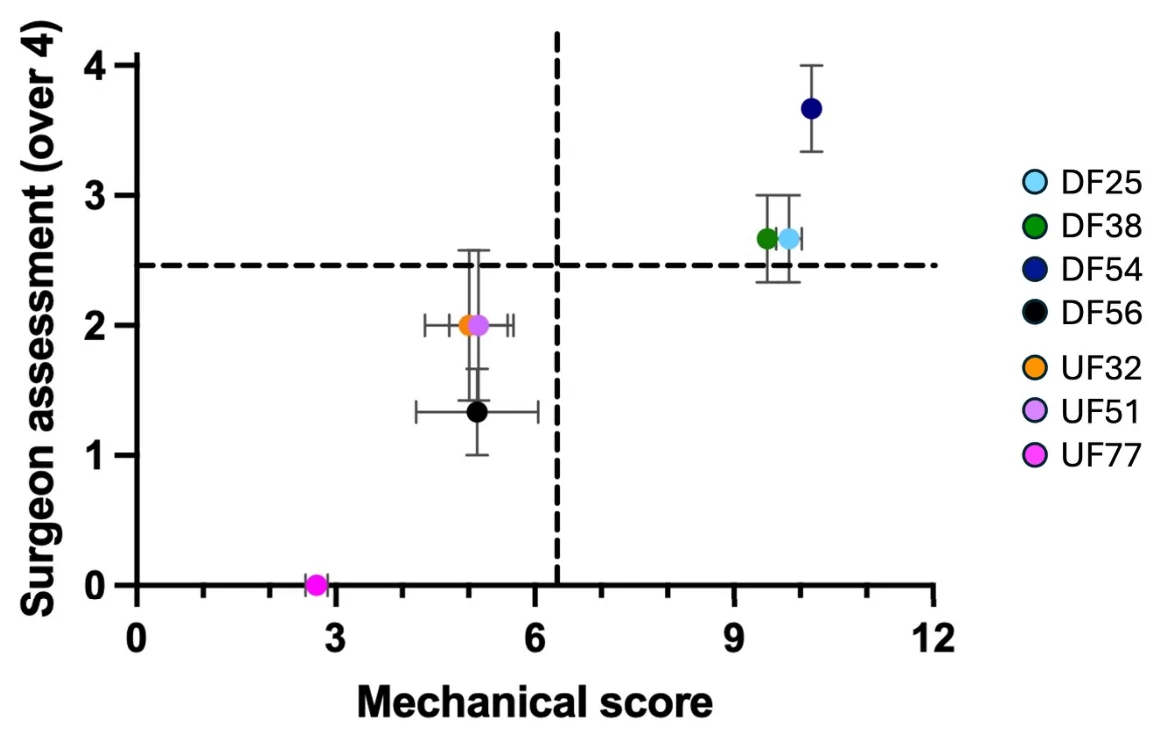
Validation graph: correlation between the calculated mechanical score and the surgeon’s evaluation of a new set of dermal (DF) and urethral (UF) stromas. Each dot represents the mean value obtained for urethral (N = 3) or dermal stromas (N = 4) of a specific cell population (n = 4 reconstructed tissue replicates per donor population). Error bars represent standard deviations. The horizontal dashed line indicates the clinical threshold score of 2.5, used to distinguish tissues considered unsuitable (<2.5) from those considered suitable (>2.5) for surgical handling. The vertical dashed line indicates the corresponding mechanical score threshold (6.35).

To further assess the applicability of the proposed scoring model, an independent validation batch of stromal samples, generated at a subsequent time point and evaluated by different experimenters, was analyzed. This batch consisted of four additional dermal (DF) populations and three additional urethral (UF) populations. For each fibroblast population, four constructs were averaged to obtain a population-level mean. Mechanical properties were measured using the puncture test, converted into property-specific scores, and incorporated into the weighted scoring equation to generate total mechanical scores. The mean total mechanical score was then calculated for each population and plotted on the classification graph against the mean score of the surgeon’s assessment (Figure 11). Validation samples were distributed within the true positive and true negative quadrants, supporting the applicability of the proposed approach for evaluating tissue-engineered stromal constructs. Specifically, 57% of the samples were classified as true negatives and 43% as true positives, while no samples were observed in the false-positive or false-negative quadrants. Thus, the mechanical classification was concordant with the surgeon assessment for all samples in this limited validation dataset.

## 4. Discussion

This study compared uniaxial tensile and puncture testing to determine whether puncture testing can serve as a practical method for assessing the mechanical properties of self-assembled tissue grafts in relation to surgeon-perceived tissue handling. The discussion focuses on the implications of these findings for establishing more objective criteria to improve the standardization of tissue-engineered graft production and evaluation.

Tissue thickness varied across cellular populations but showed no direct association with donor age (Figure 3A). This variability may arise from multiple factors known to influence fibroblast function and ECM production, including donor-related characteristics such as age, obesity, diabetes, metabolic disorders, and other comorbidities [15], as well as pre-analytical factors such as cell freezing and thawing procedures. However, as these donor characteristics were not specifically documented or analyzed in the present study, their contribution to the variability observed between donors cannot be determined. Suboptimal handling during these steps may impair cell viability, proliferation, and function, ultimately compromising ECM deposition and collagen production, which are essential for the self-assembly approach to tissue engineering used in this study [16]. This may explain the reduced thickness observed in DF36 and DF56 tissues, while the particularly low thickness of UF77 tissues may additionally be linked to donor age. Importantly, comparable thickness values were obtained across both mechanical tests, supporting an unbiased distribution of samples between testing conditions (Figure 3B).

For maximum force, substantial differences in absolute values were observed between the two methods (Figure 4A), which can be attributed to both sample preparation and loading mode. These methodological differences directly influence how mechanical stresses are transmitted through the tissue during testing. In uniaxial tensile testing, tissues are shaped into a dog-bone geometry, potentially introducing structural weakening, whereas puncture testing preserves tissue integrity. In addition, tensile loading distributes stress across the sample, while puncture applies a localized, concentrated force [17]. Despite these differences in magnitude, strong correlations were observed (Figure 4B), enabling estimation of one measurement from the other using the regression slope (~0.33). However, puncture testing may be influenced by local heterogeneity, as measurements reflect the properties of a single central point rather than the entire tissue.

Stiffness similarly exhibited substantial variability across cellular populations (Figure 5A), likely reflecting differences in fibroblast capacity to produce and organize ECM components [18]. Puncture testing yielded higher stiffness values, which can be explained by the localized concentration of stress compared to the more uniformly distributed strain in tensile testing [17]. Despite these methodological differences, both tests showed strong correlation, with high coefficients of determination and similar slopes (~0.16) (Figure 5B), supporting their comparability and their consistent relationship across both construct types.

For toughness, tensile testing generally yielded slightly lower values than puncture testing in dermal tissues (Figure 6A), whereas urethral tissues showed minimal differences between methods. Because toughness integrates the entire stress–strain response, it is less sensitive to loading mode, which may explain the closer correlation between tests. Observed differences may nevertheless reflect variations in ECM composition and organization between dermal and urethral tissues, which, although both serving structural roles, are subjected to distinct physiological constraints, such as cyclic pressure changes in the urethra [6,19,20]. While dermal tissue showed a good correlation between tests, it was weaker than for the other parameters (R^2^ ≈ 0.74).

This study compared uniaxial tensile and puncture testing to determine whether puncture testing can serve as a more practical method for assessing the mechanical quality of self-assembled tissue grafts. Given the strong correlation observed across the mechanical properties, either method could be used for correlation with surgeon-based evaluation. While both tests are destructive and preclude subsequent grafting of the tested construct, uniaxial tensile testing requires the preparation of standardized dog-bone-shaped specimens and may be more influenced by interlamellar cohesion within the self-assembled tissue. In contrast, puncture testing is simpler, faster, and better mimics the localized loading conditions associated with suturing, although it remains sensitive to local tissue heterogeneity. Furthermore, as shown in Table 2, puncture testing generally yielded lower mean coefficients of variation across the different mechanical properties in both dermal and urethral tissues, indicating lower measurement variability for most parameters compared with uniaxial tensile testing. This additional advantage further supported the selection of puncture testing for correlation with qualitative surgical assessment. In addition, in a tissue-production workflow, puncture testing could be easily performed on representative stromal constructs once they reach the appropriate stage, immediately before epithelialization. The results obtained from these representative constructs could then be used as a quality-control assessment for the corresponding tissue production batch. If the predefined mechanical criteria are met, the remaining constructs from the batch could proceed to epithelialization; if the criteria are not met, the remaining constructs could be maintained in culture for further maturation. Thus, the puncture-tested tissues would not be returned to culture, but would instead serve as representative samples for quality control of the remaining constructs.

Before performing the surgeon’s suture evaluation, it was necessary to determine whether epithelialization influences stromal mechanical properties. Although surgeons clinically manipulate epithelialized grafts, the production of epithelialized substitutes requires additional culture time, materials, and costs compared with non-epithelialized stromas. Therefore, determining whether epithelialization results in substantial changes in mechanical properties could allow subsequent experiments to be performed ahead of time, using stromas alone, thereby simplifying tissue production and reducing costs.

To address this question, puncture tests were performed on a limited number of epithelialized and non-epithelialized dermal and urethral stromas. No statistically significant differences were observed for the evaluated mechanical parameters, indicating that no substantial changes were detected following epithelialization. These results suggest that epithelialization does not significantly alter the global mechanical behavior of the constructs. Therefore, the mechanical characterization could be performed before epithelialization to obtain information on the mechanical properties of the stromal constructs, without requiring additional time and cellular resources associated with epithelialization. Consequently, non-epithelialized stromas were used for the surgeon’s suture assessment and for the remainder of the study.

To date, the literature does not provide reference threshold values for the mechanical properties that engineered urethral tissues should achieve, particularly for self-assembled tissue-engineered urethral substitutes.

In a previous study, we performed uniaxial tensile testing on our self-assembled urethral tissues and obtained maximal force values ranging from 0.1 to 1.5 N, as well as elastic modulus values ranging from 0.6 to 10 MPa^3^ [7]. However, due to the absence of relevant benchmark values for engineered urethral tissues in the literature, we relied on reported mechanical values from native human bladder tissue, using a threshold of 0.27 N, corresponding to the lower limit for maximal force, and a threshold of 0.25 MPa, corresponding to the upper limit for elastic modulus, as reference points in our analyses [21].

Since these values cannot be considered appropriate or representative threshold values for engineered urethral substitutes, this highlighted the need for a more relevant approach to mechanical characterization. Therefore, the present study was designed to improve our understanding of the mechanical behavior of tissue-engineered urethral constructs and to explore potentially relevant reference values for their mechanical characterization. To further relate mechanical characterization to the practical use of these tissues, we complemented mechanical testing with a surgeon-based assessment of tissue handling during suturing. This qualitative assessment was intended to evaluate tissue behavior under conditions that more closely reflect the surgical context.

A scoring approach was thus developed to bridge the gap between quantitative mechanical characterization and qualitative surgical perception. While mechanical properties provide objective measurements of tissue performance, the surgeon’s assessment during suturing integrates multiple aspects of tissue behavior that are difficult to directly quantify. Therefore, converting continuous mechanical measurements into an ordinal scoring system (Table 3 and Table 4) enabled direct comparison between both evaluation methods and facilitated the development of an integrated mechanical assessment model.

The introduction of the Adequacy Factor further allowed differential weighting of the evaluated mechanical parameters according to their correlation with surgical handling outcomes. Since not all mechanical properties contribute equally to the perceived quality of a tissue during manipulation and suturing, this approach prevented all parameters from having the same influence on the final score. Parameters showing stronger correlation with surgical assessment (Figure 9) contributed more substantially to the overall mechanical score, allowing the model to better capture tissue behavior relevant to surgical handling.

The quadrant-based analysis presented in Figure 10 highlighted the importance of distinguishing concordant and discordant classifications. True positive and true negative classifications indicate correlation between mechanical characterization and surgical assessment and therefore support the relevance of the proposed model. In contrast, discordant classifications provide additional insight into potential limitations of mechanical measurements alone. False-positive classifications may represent the most critical outcome because they indicate a tissue classified as mechanically suitable while being judged unsuitable by the surgeon during handling. Such cases could lead to an overestimation of tissue suitability and may have important implications for tissue selection prior to surgical implantation, potentially resulting in the loss of operating-room resources and staff time, as well as a tissue that cannot be returned to culture once brought to the surgical setting.

Conversely, false-negative classifications indicate that a tissue does not meet the predefined mechanical criteria despite favorable surgical assessment. Although these classifications still represent a discrepancy between the model and the clinical perception, they may identify tissues with adequate handling characteristics that could potentially continue to mature and improve mechanically with additional culture time. In the primary analysis presented in Figure 10, the false-negative rate was 37.5%, highlighting a limitation of the current scoring model and indicating that its predictive value for surgical suitability remains limited. Nevertheless, in the proposed tissue-production workflow, false-negative classifications may be less consequential than false-positive classifications, as they would not necessarily preclude the continued tissue-production process before epithelialization.

The classification system was initially developed at the individual construct level, whereas its applicability was subsequently assessed at the population level by averaging four constructs generated from each independent fibroblast population to account for biological variability among constructs within each population. The classification of the independent validation samples into true positive and true negative quadrants (Figure 11) supports the applicability of the proposed approach and provides preliminary evidence that the developed equation may be useful for integrating multiple mechanical parameters into a representative weighted score for tissue evaluation. This method could provide a standardized framework for the assessment of engineered tissues and potentially reduce reliance on subjective evaluation alone. However, the relatively small number of independent fibroblast populations in the validation dataset limits the generalizability of these findings and warrants further evaluation using a larger independent dataset to better establish the predictive performance of the scoring model. In addition, although the assessment was performed by a single experienced surgeon and independently observed by a non-surgeon observer, the subjective nature of the 0–4 scoring system and its calibration to a single surgeon’s assessment may limit its generalizability to other surgeons. Assessments by multiple surgeons would therefore be needed to evaluate inter-rater variability and to establish broader applicability and reproducibility of the scoring scale across surgeons.

In a practical application context, newly engineered tissues could be evaluated using puncture testing followed by calculation of the total mechanical score using the proposed equation. The resulting value could then be positioned within the classification framework (Figure 10) to guide decision-making. The vertical threshold value of 6.35 allows stratification of samples into two main regions; however, both concordant (true positive and true negative) and discordant (false positive and false negative) outcomes may be observed on either side of this threshold, as illustrated in Figure 10.

Therefore, the interpretation of individual predictions should consider the full quadrant-based classification rather than relying solely on the threshold value. Nevertheless, samples with higher mechanical scores more frequently fall within concordant regions, whereas lower scores show a greater proportion of discordant classifications. In a practical context, sub-threshold samples could be considered for extended culture to potentially improve their mechanical properties prior to implantation, whereas higher-scoring samples are more likely to meet both mechanical and surgeon-handling requirements. In this context, the proposed score could serve as a preimplantation go/no-go quality-control criterion, with a “go” classification indicating that a tissue meets the established requirements for transfer to the operating room for surgical use. Tissue release should ultimately be considered alongside other quality-control assessments, including functional properties such as permeability, for which a dedicated assessment method has previously been published [22]. Once implanted, graft functionality becomes particularly important, as its functional performance is a key determinant of its ability to fulfill its intended role and ultimately contribute to successful reconstruction.

This study has some limitations. Although a relatively large number of constructs were analyzed (approximately 104 for uniaxial tensile testing, 80 for puncture testing, and 80 for surgeon assessment), they were derived from only 13 fibroblast populations. This relatively small number of biological populations limits the statistical robustness of the findings. Increasing both the number of constructs and the number of fibroblast populations would strengthen the analysis. However, achieving this would require additional cell isolation, cell expansion, and tissue reconstruction, all of which are time-consuming and costly. In addition, the suturing assessment remains largely subjective, and although the surgeon’s assessment was performed under blind conditions and an independent non-surgeon observer was present to document and verify the assessment, formal inter-observer validation was not performed. Future studies involving multiple surgeons with varying levels of experience and performing independent assessments are needed to evaluate inter-operator variability. Furthermore, all urethral fibroblasts were derived from donors who had undergone hormonal treatments that may represent a source of biological variability [23].

The thresholds and adequacy factors were derived from the dataset to generate a first calibrated model linking mechanical measurements to surgical perception. As such, this approach should be considered exploratory, aiming to define biologically meaningful ranges that reflect surgeon-perceived tissue handling rather than universal thresholds. In this context, the proposed framework can be viewed as a simplified and fully interpretable alternative to supervised learning approaches, in which mechanical parameters are calibrated against surgeon-assigned handling scores [24]. Unlike black-box approaches [25], this methodology preserves physiological interpretability and direct clinical applicability, allowing a transparent integration of quantitative measurements with surgical expertise.

Future work should include larger datasets to support the development of predictive models, potentially using machine learning approaches, to identify the most relevant mechanical parameters and improve the accuracy of pre-graft evaluation. Further methodological validation could also benefit from combining the surgeon-based assessment with quantitative suture-retention testing. While the surgeon-based assessment was intended to capture overall tissue handling and response to suturing in a context that reflects surgical practice, suture-retention testing would provide a more objective and standardized measure of resistance to suture-related tearing. These complementary approaches could help clarify the relationship between puncture mechanical properties, resistance to suture-related tearing, and overall surgical handling.

Expanding the mechanical characterization of engineered tissues would also be valuable through the inclusion of additional tests, such as biaxial tensile testing to better replicate multidirectional in vivo loading conditions [26], and tear resistance tests to measure tear propagation after initial damage [27].

Further investigations could also focus on the intrinsic differences between tissues associated with low and high surgeon suture scores, including analyses of tissue architecture, inter-sheet cohesion, structural properties, and ECM composition, to better understand the determinants of favorable surgical handling. Such studies may help identify the microstructural features that influence suture retention and tissue tearing behavior. Finally, the development and integration of non-destructive mechanical or imaging-based approaches would be of particular interest, as they could enable longitudinal assessment of tissue maturation within the same constructs without compromising tissue integrity, thereby improving monitoring efficiency and providing a more comprehensive characterization of tissue development over time.

## 5. Conclusions

This study demonstrates that uniaxial tensile and puncture testing, although yielding different absolute values, provide comparable assessments for most mechanical properties of tissues reconstructed from the same populations. By integrating quantitative mechanical measurements obtained through puncture testing with surgeon-based evaluation, a weighted mechanical scoring model was developed to relate objective tissue characterization to surgical handling assessment.

The classification of independent validation samples provides proof-of-concept support for the proposed approach and suggests that the model may be useful for integrating multiple mechanical parameters into an objective framework for tissue evaluation during production. Further validation using larger and independent datasets, including assessments by multiple surgeons, will be necessary to establish the generalizability and reproducibility of the scoring model. Ultimately, this approach could contribute to the development of standardized quality-control strategies for tissue-engineered grafts and support their future clinical translation.

## Figures and Tables

**Table 1:** Characteristics of fibroblast populations according to tissue origin, sex, and age. Dermal fibroblasts (DF), Keratinocytes (K), urethral fibroblasts (UF) and urethral urothelial cells (UUC).

Fibroblast Population	Epithelial Cell Population	Origin	Age (Years Old)	Sex
DF25	K25	skin	25	female
DF34	K34	skin	34	female
DF36	K36	skin	36	female
DF38	K38	skin	38	female
DF51	K51	skin	51	female
DF54	K54	skin	54	female
DF56	K56	skin	56	female
UF22	UUC22	urethra	22	transgender woman
UF32	UUC32	urethra	32	transgender woman
UF38	UUC38	urethra	38	transgender woman
UF51	UUC51	urethra	51	transgender woman
UF72	UUC72	urethra	72	transgender woman
UF77	UUC77	urethra	77	transgender woman

**Table 2:** Mean coefficients of variation in mechanical properties assessed by uniaxial tensile and puncture testing for dermal (DF) and urethral (UF) constructs.

	Maximum Force		Stiffness		Toughness	
	Tensile Test	Puncture Test	Tensile Test	Puncture Test	Tensile Test	Puncture Test
DF	22.69	19.89	36.85	16.49	25.77	24.63
UF	25.50	28.92	34.10	23.69	45.55	38.70

**Table 3:** Scoring criteria for mechanical properties based on predefined value ranges.

Score	1	2	3	4
Thickness (μm)	0 to 50	50 to 100	100 to 200	200+
Maximum force (N)	0 to 0.4	0.4 to 0.8	0.8 to 1.6	1.6+
Stiffness (mN/mm)	0 to 400	400 to 800	800 to 1600	1600+
Toughness (energy)	0 to 0.35	0.35 to 0.7	0.7 to 1.4	1.4+

**Table 4:** Measured mechanical properties, corresponding scores and surgeon assessment for tested tissue samples. Dermal (DF) and urethral (UF) constructs.

	Thickness (μm)		Maximum Force (N)		Stiffness (mN/mm)		Toughness (Energy)			
Factor	0.667		0.625		0.708		0.542			
Tissue	Value	Associated score	Value	Associated score	Value	Associated score	Value	Associated score	Total score ∑ (scores × IF)	Surgeon assessment
UF22-1	92.33	2	0.35	1	1187.22	3	0.869	3	5.708	3
UF22-2	112.88	3	0.37	1	1229.45	3	0.828	3	6.375	4
UF22-3	93.50	2	0.42	2	1184.61	3	0.927	3	6.333	2
UF22-4	83.88	2	0.34	1	1079.82	3	0.772	3	5.708	3
UF38-1	143.50	3	0.27	1	778.13	2	0.637	2	5.125	4
UF38-2	111.50	3	0.48	2	743.60	2	0.533	2	5.750	3
UF38-3	120.50	3	0.36	1	874.05	3	0.642	2	5.833	2
UF38-4	138.38	3	0.36	1	753.49	2	0.538	2	5.125	2
UF72-1	105.33	3	0.84	3	1476.19	3	1.269	3	7.625	4
UF72-2	124.50	3	0.86	3	1719.97	4	1.531	4	8.875	4
UF72-3	91.13	2	0.54	2	1925.45	4	1.654	4	7.583	3
UF72-4	115.33	3	0.70	2	1854.75	4	1.836	4	8.250	3
DF34-1	181.17	3	1.22	3	409.77	2	0.239	1	5.833	3
DF34-2	220.83	4	1.21	3	400.83	2	0.211	1	6.500	4
DF34-3	154.83	3	1.26	3	398.78	1	0.336	1	5.125	4
DF34-4	152.75	3	1.12	3	341.18	1	0.240	1	5.125	3
DF36-1	182.67	3	0.89	3	313.21	1	0.178	1	5.125	2
DF36-2	64.75	2	0.77	2	425.77	2	0.373	2	5.083	3
DF36-3	78.25	2	0.93	3	336.98	1	0.238	1	4.458	3
DF36-4	73.13	2	0.79	2	379.33	1	0.260	1	3.833	1
DF51-1	255.33	4	1.71	4	988.78	3	0.432	2	8.375	4
DF51-2	167.00	3	2.00	4	985.26	3	0.477	2	7.708	4
DF51-3	219.00	4	2.12	4	1064.80	3	0.477	2	8.375	4
DF51-4	191.38	3	2.24	4	695.28	2	0.284	1	6.458	4

## Data Availability

All data are available upon reasonable request from the corresponding author.

## Supplementary Materials

The following supporting information can be downloaded at https://www.mdpi.com/article/10.3390/bioengineering13091051/s1, Figure S1. Maximum force of stromal tissues with epithelium; Figure S2. Stiffness of stromal tissues with epithelium; Figure S3. Toughness of stromal tissues with epithelium; Figure S4. Maximum force of stromas and stromal tissues with epithelium; Figure S5. Stiffness of stromas and stromal tissues with epithelium; Figure S6. Toughness of stromas and stromal tissues with epithelium; Table S1. Measured mechanical properties and corresponding scores for the validation tissue sample; Figure S7. Representative histological sections of dermal stromas and dermal stromas overlaid with epithelium generated using fibroblasts from different donors.
