# Supplementary material for: From Bench to Operating Room: A Comparative Study of Tensile and Puncture Tests in Self-Assembled Constructs with Clinical Feedback
Source: Bioengineering (Basel). 2026 Sep 10;13(9):1051. doi: 10.3390/bioengineering13091051 (PMC13603881; doi:10.3390/bioengineering13091051)
Supplement: Supplementary file 1 [file bioengineering-13-01051-s001.zip › bioengineering-4520155-supplementary.pdf]

Supplementary material

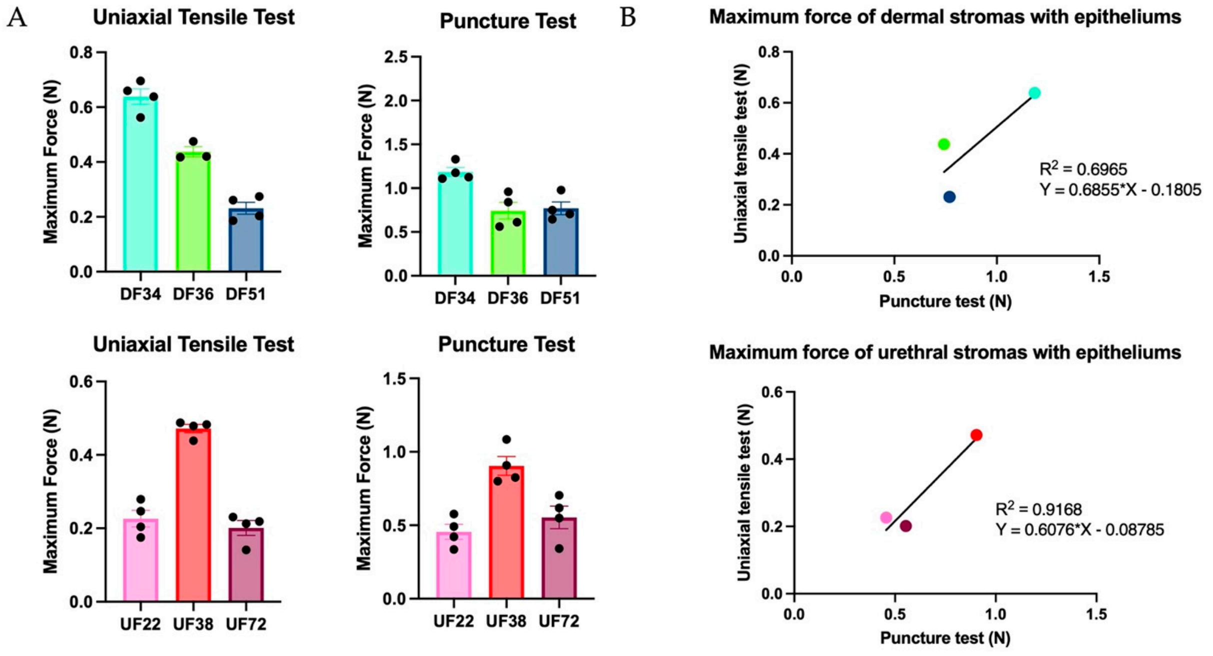

Figure S1. Maximum force of stromal tissues with epithelium. **A.** Mean maximum forces (in newtons) of the different dermal (DF) and urethral (UF) populations used for the uniaxial tensile test and the puncture test. Each column represents a stromal population with epithelium, ranked according to donor age. **B.** Correlation between the mean maximum forces obtained from the two tests for dermal and urethral stromal tissues with epithelium.

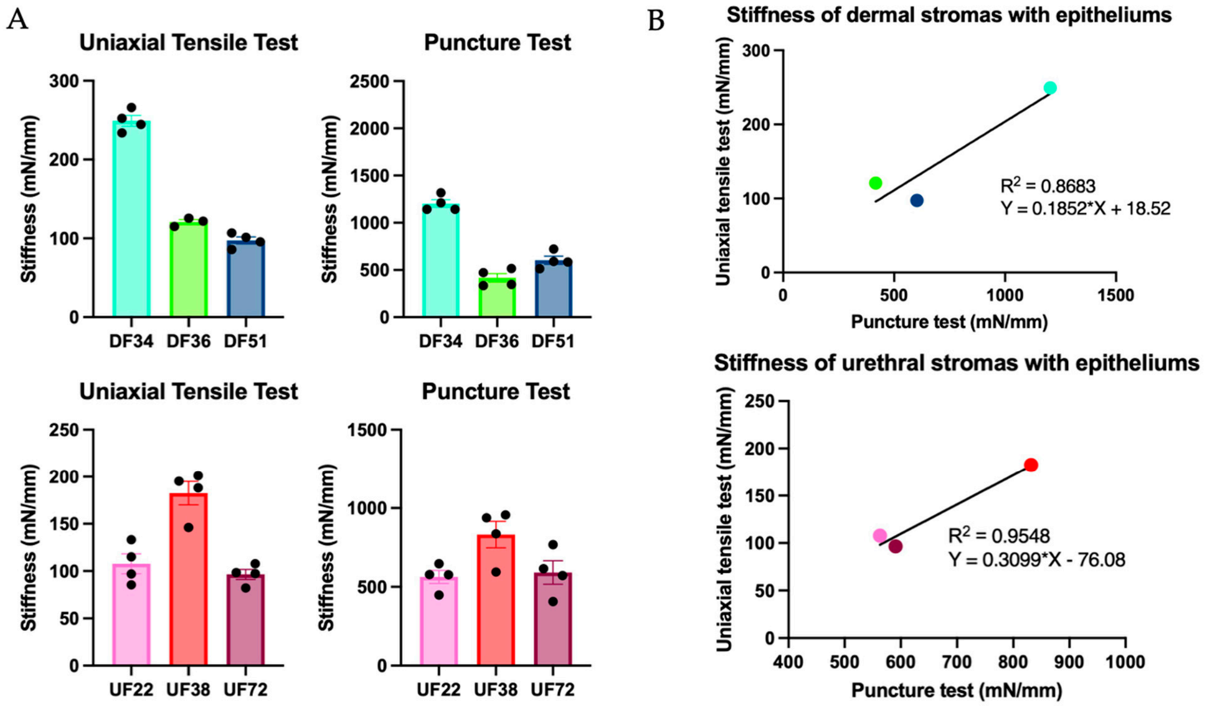

Figure S2. Stiffness of stromal tissues with epithelium. **A.** Mean stiffness (in millinewtons/millimeters) of the different dermal (DF) and urethral (UF) populations used for the uniaxial tensile test and the puncture test. Each column represents a stromal population with epithelium, ranked according to donor age. **B.** Correlation between the mean stiffness obtained from the two tests for dermal and urethral stromal tissues with epithelium.

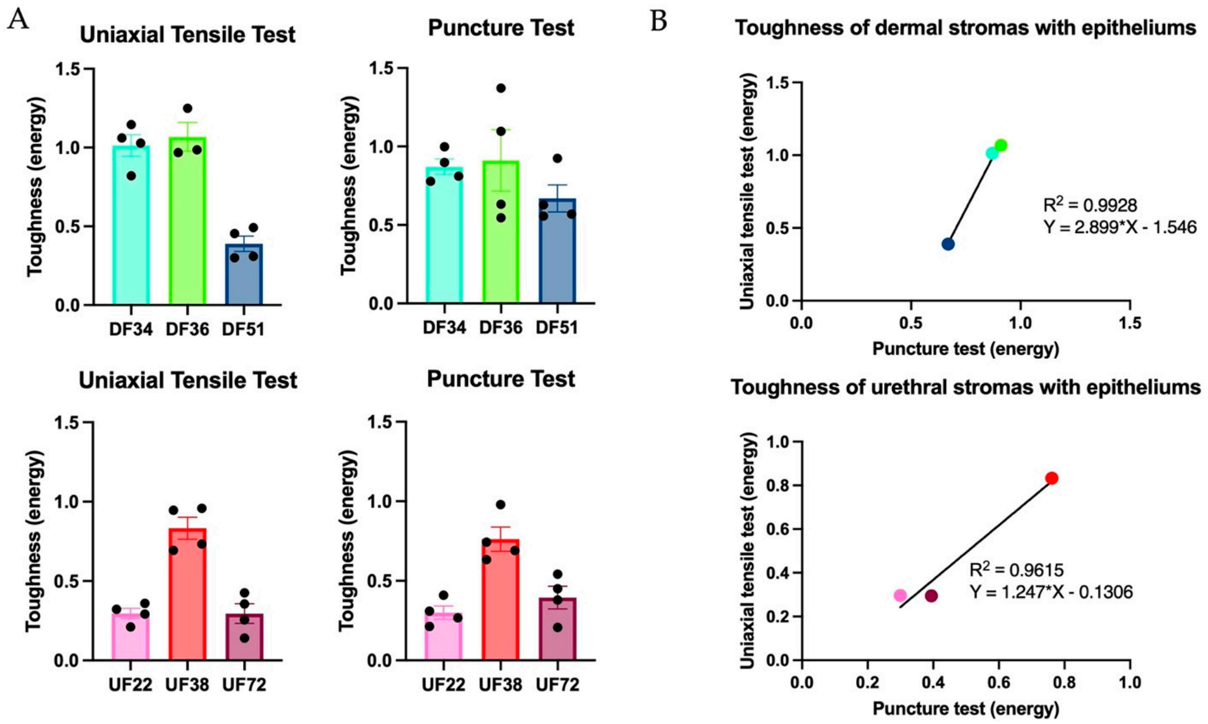

Figure S3. Toughness of stromal tissues with epithelium. **A.** Mean toughness (in energy) of the different dermal (DF) and urethral (UF) populations used for the uniaxial tensile test and the puncture test. Each column represents a stromal population with epithelium, ranked according to donor age. **B.** Correlation between the mean toughness obtained from the two tests for dermal and urethral stromal tissues with epithelium.

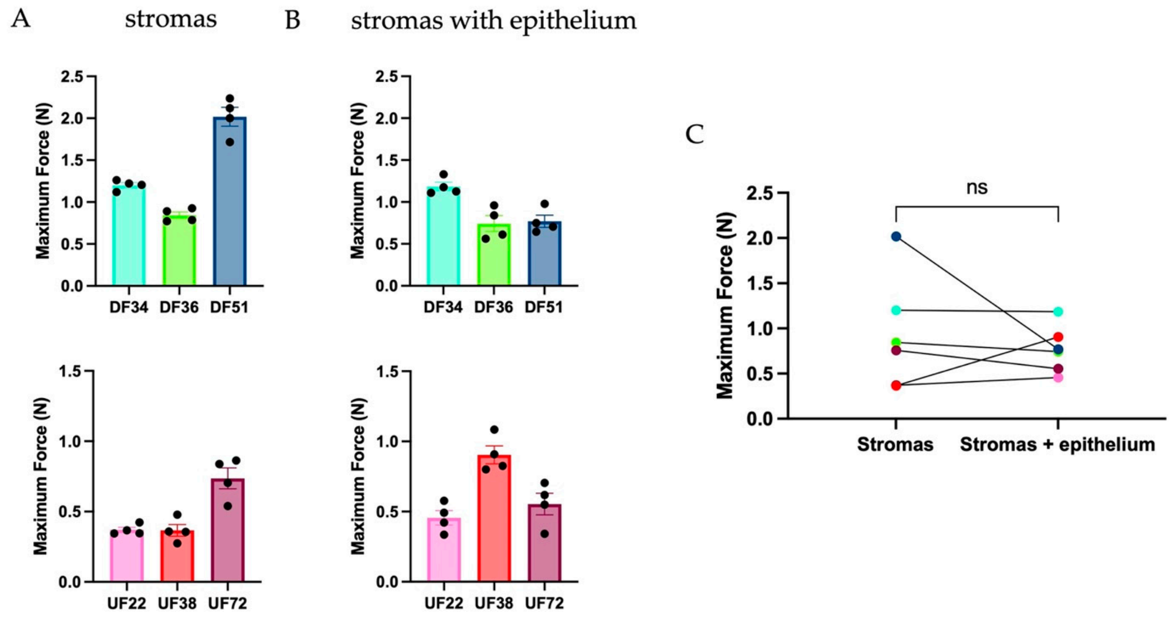

Figure S4. Maximum force of stromas and stromal tissues with epithelium. **A.** Mean maximum force (in newtons) of the different dermal (DF) and urethral (UF) stromal populations used for the uniaxial tensile test and the puncture test. Each column represents a stromal population, categorized according to donor age. **B.** Mean maximum force of the different dermal (DF) and urethral (UF) populations used for the uniaxial tensile test and the puncture test. Each column represents a stromal population with epithelium, ranked according to donor age. **C.** Correlation between the mean maximum force obtained from the stromas and the stromal tissues with epithelium.

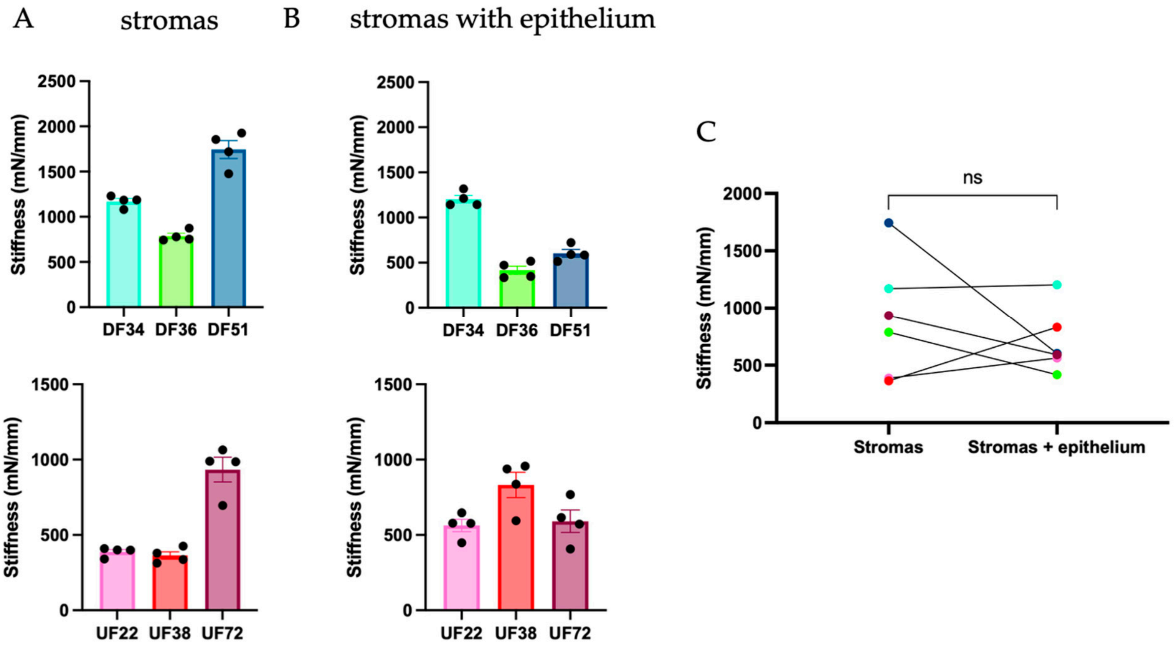

Figure S5. Stiffness of stromas and stromal tissues with epithelium. **A.** Mean stiffness (in mN/mm) of the different dermal (DF) and urethral (UF) stromal populations used for the uniaxial tensile test and the puncture test. Each column represents a stromal population, categorized according to donor age. **B.** Mean stiffness of the different dermal (DF) and urethral (UF) populations used for the uniaxial tensile test and the puncture test. Each column represents a stromal population with epithelium, ranked according to donor age. **C.** Correlation between the stiffness obtained from the stromas and the stromal tissues with epithelium.

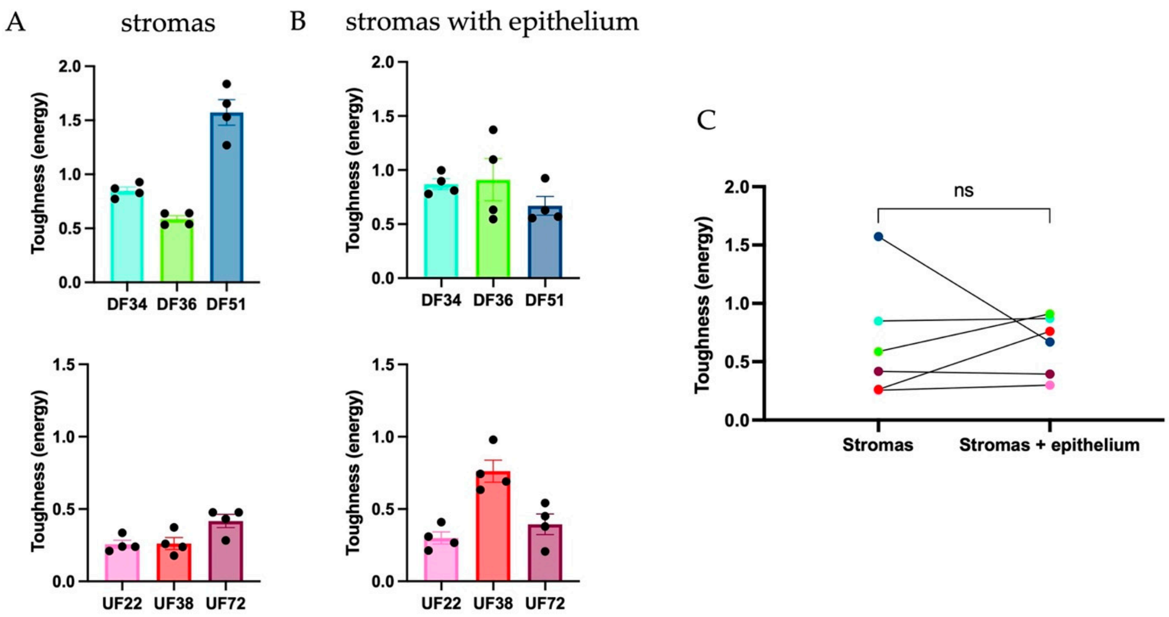

Figure S6. Toughness of stromas and stromal tissues with epithelium. **A.** Mean toughness (in energy) of the different dermal (DF) and urethral (UF) stromal populations used for the uniaxial tensile test and the puncture test. Each column represents a stromal population, categorized according to donor age. **B.** Mean toughness of the different dermal (DF) and urethral (UF) populations used for the uniaxial tensile test and the puncture test. Each column represents a stromal population with epithelium, ranked according to donor age. **C.** Correlation between the toughness obtained from the stromas and the stromal tissues with epithelium.

Table S1. Measured mechanical properties and corresponding scores for the validation tissue samples. Dermal (DF) and urethral (UF) constructs.

	Thickness (µm)		Maximum force (N)		Stiffness (mN/mm)		Toughness (energy)			
Factor	0.667		0.625		0.708		0.542			
Tissue	Value	Associated score	Value	Associated score	Value	Associated score	Value	Associated score	Total score ∑ (scores x IF)	Mean
DF25-1	133.90	3	2.64	4	2236.89	4	2.21	4	9.50	9.83
DF25-2	224.90	4	4.07	4	3223.51	4	3.24	4	10.17	
DF25-3	161.30	3	2.07	4	1829.13	4	1.58	4	9.50	
DF25-4	230.70	4	3.17	4	2474.59	4	2.53	4	10.17	
DF38-1	150.60	3	2.68	4	2127.01	4	2.08	4	9.50	9.50
DF38-2	149.10	3	2.14	4	1811.16	4	1.56	4	9.50	
DF38-3	185.00	3	2.46	4	1950.73	4	1.99	4	9.50	
DF38-4	190.00	3	2.83	4	2793.37	4	1.90	4	9.50	
DF54-1	238.80	4	3.22	4	2797.24	4	2.39	4	10.17	10.17
DF54-2	245.50	4	2.25	4	2203.76	4	1.53	4	10.17	
DF54-3	245.40	4	4.09	4	2968.83	4	3.47	4	10.17	
DF54-4	247.90	4	3.33	4	2937.14	4	2.34	4	10.17	
DF56-1	80.80	2	0.96	3	927.57	3	0.65	2	6.42	5.13
DF56-2	38.60	1	0.46	2	483.50	2	0.26	1	3.88	
DF56-3	92.40	2	1.01	3	911.35	3	0.73	3	6.96	
DF56-4	34.90	1	0.33	1	521.20	2	0.13	1	3.25	
UF32-1	60.60	2	0.25	1	345.62	1	0.11	1	3.21	5.01
UF32-2	207.70	4	0.60	2	558.71	2	0.41	2	6.42	
UF32-3	198,1	3	0,49	2	492,87	2	0,33	1	5,21	
UF32-4	158,1	3	0,54	2	665,11	2	0,29	1	5,21	
UF51-1	140,6	3	0,69	2	526,79	2	0,62	2	5,75	5,15
UF51-2	177,8	3	0,53	2	531,51	2	0,34	1	5,21	
UF51-3	103,4	3	0,16	1	162,46	1	0,11	1	3,88	
UF51-4	183,2	3	0,66	2	559,06	2	0,50	2	5,75	
UF77-1	76,5	2	0,03	1	34,63	1	0,01	1	3,21	2,71
UF77-2	20,3	1	0,03	1	33,74	1	0,02	1	2,54	
UF77-3	23,1	1	0,01	1	14,80	1	0,00	1	2,54	
UF77-4	33,6	1	0,03	1	37,92	1	0,01	1	2,54	

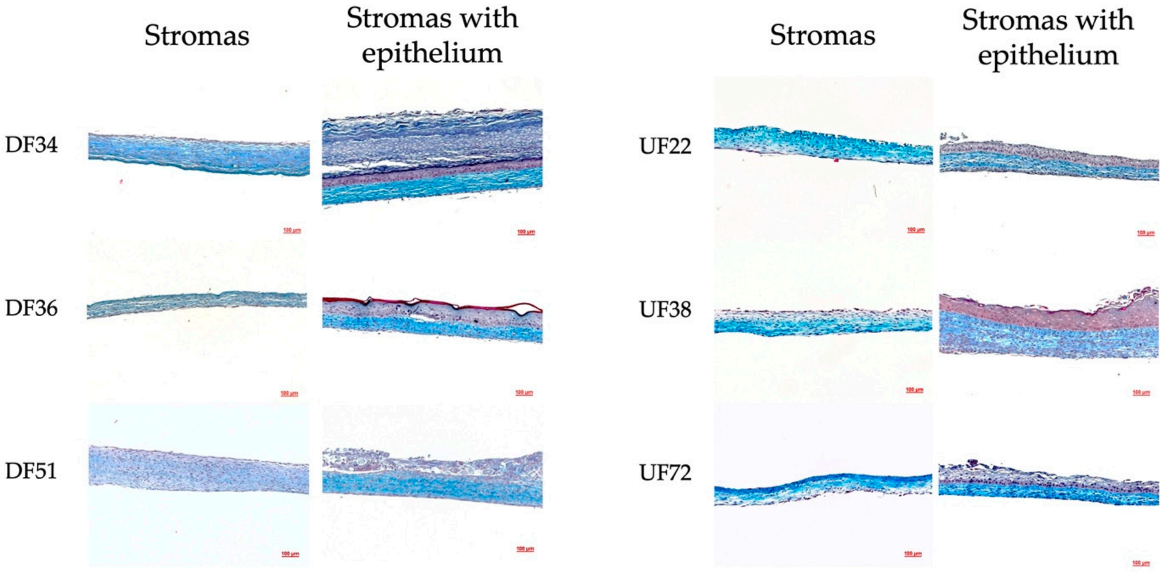

Figure S7. Representative histological sections of dermal stromas and dermal stromas overlaid with epithelium generated using fibroblasts from different donors. Sample identifiers (DF34, DF36, DF51, UF22, UF38, and UF72) correspond to the donor-derived fibroblast cell lines, where the letters indicate the cell line and the number indicates the donor's age in years (e.g., DF34 = dermal fibroblasts from a 34-year-old donor). Scale bar = 100 μm.
